# Tau Overexpression Causes Cardiac Hypertrophy by Disrupting Mitochondrial Homeostasis

**DOI:** 10.3390/ijms27188075

**Published:** 2026-09-11

**Authors:** Jingping Xu, Yujia Xing, Qiqi Liu, Tianle Wang, Wenjia Liu, Xiao Yang, Zhenhua Li, Jian Wang

**Affiliations:** 1 State Key Laboratory of Medical Proteomics, National Center for Protein Sciences (Beijing), Beijing 102206, Chinayangx@bmi.ac.cn (X.Y.); 2 College of Life Sciences, Hebei University, Baoding 071002, China

**Keywords:** Tau, cardiac hypertrophy, mitochondrial homeostasis, therapeutic target

## Abstract

Tau is classically defined as a microtubule-associated protein whose dysfunction underpins neurodegenerative disorders. However, its role in cardiac pathophysiology remains elusive despite recent evidence of its cardiac expression. Here, we report that a ~55 kDa Tau-immunoreactive species, which is commonly found in the nervous system, is markedly upregulated in isoproterenol-induced hypertrophic murine hearts, suggesting that this stress-inducible species is associated with maladaptive cardiac remodeling. Forced Tau expression was sufficient to induce hypertrophic growth in cultured cardiomyocytes. Cardiomyocyte-restricted Tau overexpression in transgenic mice recapitulated the progressive trajectory from hypertrophy to overt heart failure. Mechanistically, Tau overexpression triggered excessive mitochondrial fission, leading to mitochondrial dysfunction evidenced by reduced mitochondrial membrane potential, elevated reactive oxygen species, diminished activity of respiratory chain complexes I and IV, and decreased intracellular ATP. Pharmacological inhibition of mitochondrial fission with Mdivi-1 attenuated Tau overexpression-induced cardiomyocyte hypertrophy; taken together, our gain-of-function evidence identifies the ~55 kDa Tau-immunoreactive species as a stress-associated factor that disrupts mitochondrial dynamics to promote cardiac remodeling, and suggests Tau as a potential therapeutic target for cardiac diseases.

## 1. Introduction

As a metabolically active organ, the adult heart exhibits one of the highest ATP demands in the body and relies primarily on mitochondrial fatty acid β-oxidation to sustain continuous contraction [1]. An integrated mitochondrial quality control (MQC) network—encompassing proteostasis, biogenesis, fusion/fission dynamics, and mitophagy—ensures organelle fitness and metabolic efficiency [2,3]. In cardiovascular disease, the MQC network becomes disrupted. In particular, excessive mitochondrial fission and impaired mitophagy drive mitochondrial fragmentation, bioenergetic failure, and oxidative stress, all of which contribute to pathological remodeling and heart failure [4,5,6]. Genetic studies have further linked impaired mitochondrial homeostasis to cardiomyopathies, underscoring the pathogenic significance of mitochondrial dysregulation in the heart [7,8]. Despite this, the cardiomyocyte-intrinsic factors that couple pathological stress to maladaptive mitochondrial dynamics remain largely undefined.

Emerging evidence suggests unexpected molecular parallels between neurodegenerative and cardiovascular diseases, where proteins traditionally considered brain-specific may also exert pathogenic roles in cardiac pathology [9]. One candidate linking these two organ systems is the microtubule-associated protein Tau (encoded by Mapt). Classically studied in the nervous system, Tau exists as multiple lower-molecular-weight isoforms (approximately 45–65 kDa) generated by alternative splicing, as well as a high-molecular-weight (HMW) isoform (110 kDa) that incorporates an additional large exon [10,11]. Canonically, Tau governs axonal transport, cell polarity, and neural transmission; however, its pathological aggregation is a hallmark of tauopathies such as Alzheimer’s disease and frontotemporal dementia [12,13]. In these disorders, aberrant Tau perturbs mitochondrial trafficking, bioenergetics, and dynamics, thereby amplifying neurotoxicity. Emerging evidence suggests Tau is not strictly brain-restricted: a 2023 study detected the HMW Tau isoform in both murine and human myocardium and implicated it in diastolic dysfunction [14], raising the possibility that Tau-mediated mitochondrial dysfunction, a central feature of neurodegeneration, may also operate in the heart. Nevertheless, whether lower-molecular-weight Tau species are induced in diseased hearts, actively contribute to cardiac remodeling, or mechanistically interface with mitochondrial dynamics in cardiomyocytes remains poorly understood.

Here, we reveal a ~55 kDa Tau-immunoreactive species as a driver of pathological cardiac remodeling. Forced Tau expression is sufficient to induce hypertrophy in cultured cardiomyocytes, and cardiomyocyte-specific Tau overexpression in mice recapitulates cardiac hypertrophy that progresses to heart failure. Mechanistically, Tau overexpression promotes excessive mitochondrial fission, leading to loss of membrane potential, ROS accumulation, impaired respiratory chain activity, and ATP depletion. Pharmacological inhibition of mitochondrial fission by Mdivi-1 attenuates Tau overexpression-induced hypertrophy, supporting a functional link between Tau-associated mitochondrial fragmentation and cardiac remodeling. Collectively, our gain-of-function evidence demonstrates that Tau overexpression is sufficient to destabilize mitochondrial homeostasis through the fission axis and promote cardiac pathology, and positions the Tau–mitochondrial dynamics interface as a potential therapeutic axis at the crossroads of neurodegenerative and cardiovascular disease.

## 2. Results

### 2.1. Tau Is Upregulated in Isoproterenol-Induced Hypertrophic Murine Hearts

**Figure 1:**
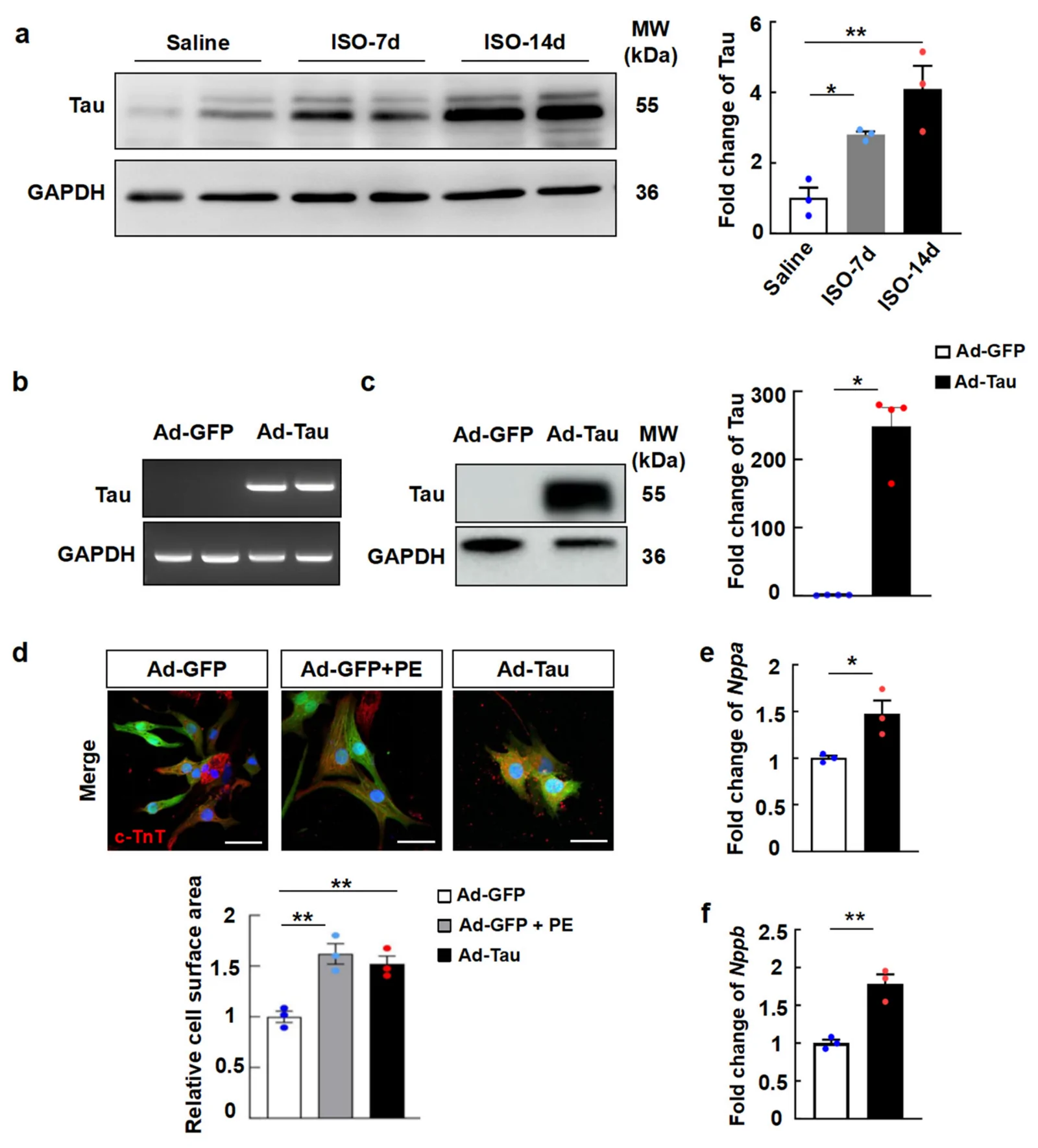
Tau overexpression induces hypertrophic growth of cardiomyocytes. (**a**) Representative Western blot (**left**) and quantification (**right**) of Tau protein expression in heart tissues from saline- or ISO-treated mice (C57BL/6J) (7 or 14 days, n = 3 mice per group). Statistical analysis was performed using one-way ANOVA followed by Dunnett’s multiple comparison test versus the saline group. (**b**) RT-PCR analysis of Tau mRNA expression levels in cardiomyocytes. (**c**) Representative Western blot (**left**) and quantification (**right**) of Tau protein in Ad-GFP or Ad-Tau-transduced NRVMs (n = 4 independent experiments). Statistical significance was determined by the Mann–Whitney U test. (**d**) Representative immunofluorescence images of cardiomyocytes immunostained for α-Actinin. Scale bar, 50 µm. Bottom: Quantification of the relative cell surface area of ventricular cardiomyocytes (n = 3 independent experiments). Statistical analysis was performed using one-way ANOVA followed by Dunnett’s multiple comparison test versus the Ad-GFP group. (**e**,**f**) Real-time PCR analyses of *Nppa* and *Nppb* in cardiomyocytes (n = 3 independent experiments); statistical significance was determined by Student’s *t*-test. Data are presented as mean ± SEM, * *p* < 0.05, ** *p* < 0.01.

To determine whether Tau responds to pathological stress in the heart, we examined Tau expression in a mouse model of cardiac remodeling induced by intraperitoneal injection of isoproterenol (ISO) in 2-month-old male C57BL/6J mice for 7 or 14 consecutive days. Compared to control mice treated with saline, Tau protein levels were markedly upregulated in cardiac tissues of ISO-treated mice at day 7 and continued to increase through day 14 (Figure 1a), suggesting that Tau may be involved in the onset and progression of cardiac remodeling.

### 2.2. Tau Overexpression Induces Hypertrophic Growth of Cardiomyocytes In Vitro

To determine whether Tau directly drives cardiomyocyte hypertrophy, we overexpressed Tau in primary neonatal rat cardiomyocytes via adenoviral transduction (Ad-Tau), with Ad-GFP as a control. Efficient Tau overexpression was confirmed at both mRNA and protein levels (Figure 1b,c). At 48 hours post-infection, cardiomyocytes were immunostained for cTnT to assess morphological changes. Cardiomyocytes treated with phenylephrine (PE) or infected with Ad-Tau exhibited significantly larger cell surface areas than Ad-GFP controls (Figure 1d). Consistently, the fetal cardiac genes natriuretic peptide A (*Nppa*) and natriuretic peptide B (*Nppb*) were upregulated in Ad-Tau-treated cardiomyocytes (Figure 1e,f). These results suggest that Tau overexpression is sufficient to induce hypertrophic growth in cardiomyocytes.

### 2.3. Cardiomyocyte-Specific Overexpression of Tau Induces Cardiac Hypertrophy and Cardiac Dysfunction

**Figure 2:**
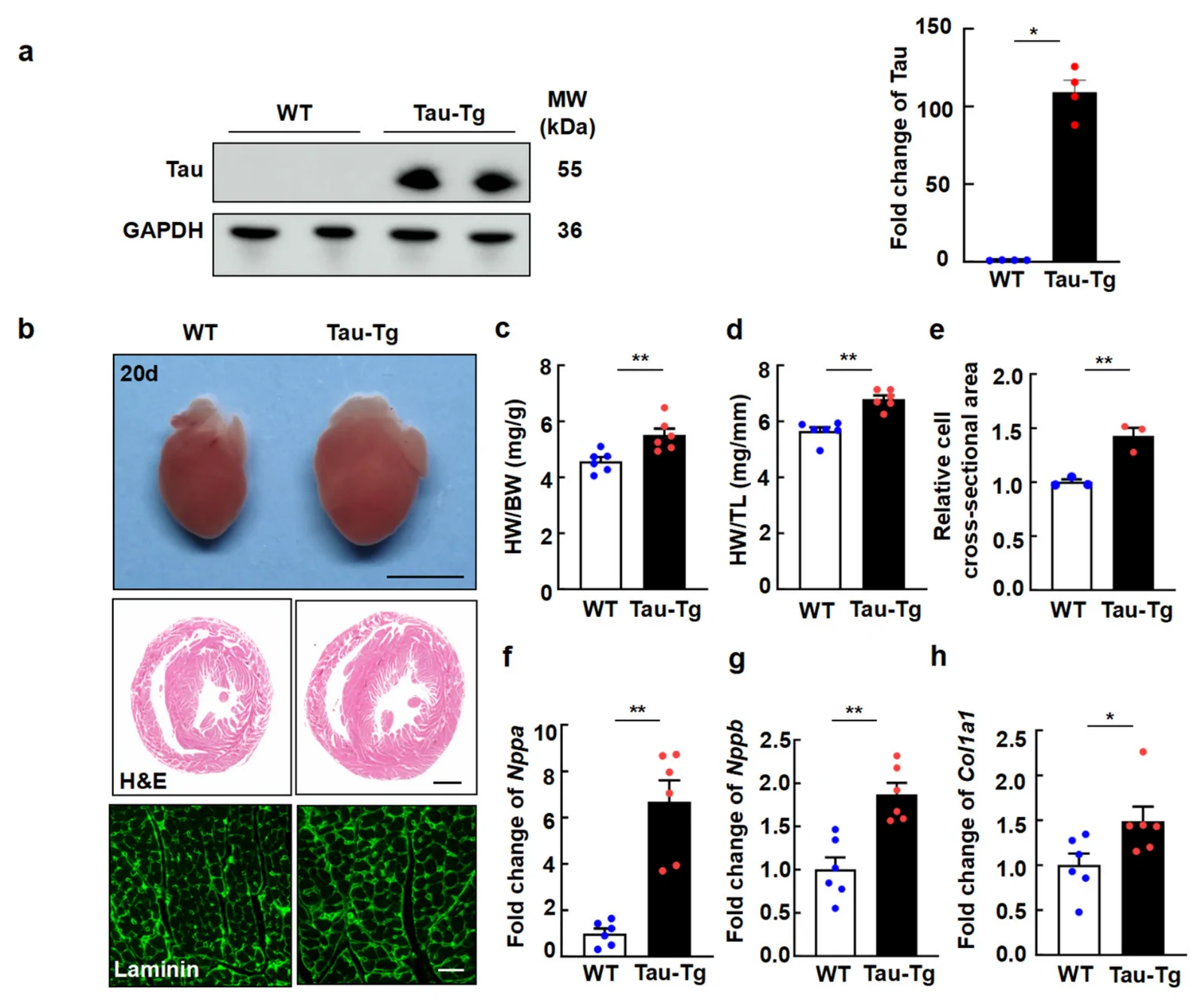
Cardiomyocyte-specific Tau overexpression transgenic mice develop cardiac hypertrophy. (**a**) Representative Western blot (**left**) and quantification (**right**) of Tau protein in hearts from WT and Tau-Tg mice (FVB/N) at postnatal day 20 (n = 4 mice per group). Statistical significance was determined by a two-tailed unpaired Student’s *t*-test. (**b**) Gross and histological morphology of the hearts from different groups of mice. (**Upper**): gross morphology analysis of hearts, Scale bar: 5 mm; (**Middle**): hematoxylin and eosin (H&E)-stained cardiac sections. Scale bar: 1 mm; (**Bottom**): laminin staining of heart tissue sections, Scale bar: 50 µm. (**c**,**d**) Quantification of heart weight to body weight (HW/BW) and tibia length (HW/TL) ratios in different groups of mice (n = 6 mice per group). For (HW/BW), statistical significance was determined by a two-tailed unpaired Student’s *t*-test; for (HW/TL), statistical significance was determined by the Mann–Whitney U test. (**e**) Quantification of relative cell cross-sectional areas (n = 3 mice per group). (**f**–**h**) Real-time PCR analysis of *Nppa*, *Nppb*, and *Col1a1* in the heart tissues of mice (n = 6 mice per group). For *Nppa* and *Nppb*, statistical significance was determined by two-tailed unpaired Student’s *t*-test; for *Col1a1*, statistical significance was determined by Mann–Whitney U test. Data are presented as mean ± standard error (SEM); * *p* < 0.05, ** *p* < 0.01.

To investigate the role of Tau in cardiac remodeling in vivo, we generated transgenic mice expressing mouse Tau cDNA under the control of the α-MHC promoter (Tau-Tg). Western blot analysis confirmed successful Tau overexpression in the transgenic hearts (Figure 2a). Tau transgenic mice were viable and fertile, but developed significant cardiac hypertrophy by 20 days of age (Figure 2b), as evidenced by increased heart weight-to-body weight (HW/BW) and heart weight-to-tibia length (HW/TL) ratios compared to littermate controls (Figure 2c,d). Hematoxylin and eosin (H&E) staining and laminin immunostaining of heart cross-sections revealed thickened left ventricular (LV) walls and enlarged cardiomyocytes in the transgenic mice (Figure 2b), with quantitative analysis confirming an increased cross-sectional area (Figure 2e). Additionally, fetal genes such as *Nppa* and *Nppb* were significantly upregulated (Figure 2f,g), and the profibrotic gene *Col1a1* showed early elevation despite the absence of overt interstitial fibrosis at this stage (Figure 2h).

**Figure 3:**
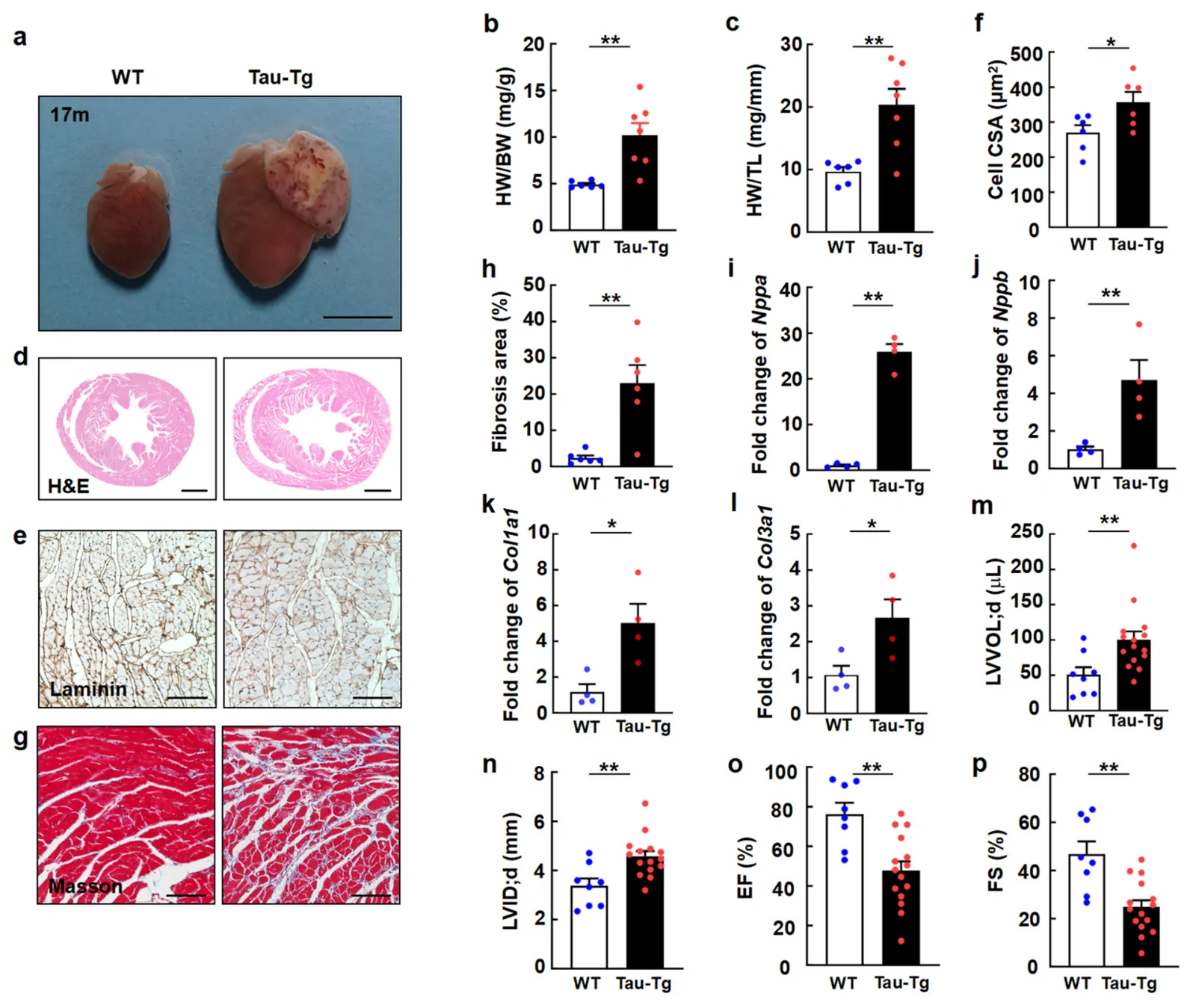
Tau overexpression promotes progressive heart failure. (**a**) Gross morphology of the heart from 17-month-old control and Tau-Tg mice.(FVB/N). Scale bar, 5 mm. (**b**,**c**) Ratios of HW/BW and HW/TL in control and Tau-Tg mice (Control mice: n = 6; Tau-Tg mice: n = 7). Statistical significance was determined by a two-tailed unpaired Student’s *t*-test. (**d**) Hematoxylin and eosin (H&E)-stained cardiac sections from different groups of mice. Scale bar, 2 mm. (**e**) Laminin immunostaining of heart tissue sections. Scale bar, 50 µm. (**f**) Quantification of cell cross-sectional areas (n = 6 mice per group). Statistical significance was determined by a two-tailed unpaired Student’s *t*-test. (**g**) Masson staining of heart tissue sections. Scale bar, 100 µm. (**h**) Quantification of fibrosis areas in hearts (n = 6 mice per group). Statistical significance was determined by a two-tailed unpaired Student’s *t*-test. (**i**–**l**) Real-time PCR analysis of *Nppa*, *Nppb*, *Col1a1*, and *Col3a1* mRNA levels in heart tissues of mice (n = 4 mice per group). Statistical significance was determined by a two-tailed unpaired Student’s *t*-test. (**m**–**p**) Echocardiographic measurement of LVVOL; d, LVID; d, EF and FS in different groups of mice (Control mice: n = 8; Tau-Tg mice: n = 15). For LVVOL; d, statistical significance was determined by the Mann–Whitney U test; For LVID; d, EF, and FS, statistical significance was determined by a two-tailed unpaired Student’s *t*-test. Data are presented as mean ± SEM, * *p* < 0.05, ** *p* < 0.01.

To assess the temporal progression of Tau-induced pathology, we examined Tau-Tg mice at 10 and 17 months of age. By 10 months, transgenic hearts exhibited progressive left ventricular enlargement and wall thickening (Figure S1). At 17 months, these changes culminated in pronounced cardiac dilation, severe wall thickening, extensive interstitial fibrosis, and persistent upregulation of fetal and profibrotic genes (*Nppa*, *Nppb*, *Col1a1*, and *Col3a1*) (Figure 3a–l).

To assess the impact of Tau overexpression on cardiac function, echocardiograms were performed at three different time points (Table S1). At 2 months of age, LV dimensions and systolic function did not differ significantly between Tau-Tg and control mice. By 10 months, Tau transgenic mice displayed increased LV posterior wall thickness at end-diastole (LVPW; d), LV internal dimension at end-systole (LVID; d), and LV volume at end-diastole (LVVOL; d) (Figure S1). By 17 months, Tau transgenic mice exhibited further increases in left ventricular wall thickness and left ventricular volume, along with significantly reduced left ventricular ejection fraction (EF%) and fractional shortening (FS%) (Figure 3m–p, Table S1). Collectively, these results demonstrate that cardiomyocyte-specific Tau overexpression in vivo drives the development of cardiac hypertrophy that progressively deteriorates into heart failure.

### 2.4. Tau Overexpression Exacerbates ISO-Induced Cardiac Remodeling

**Figure 4:**
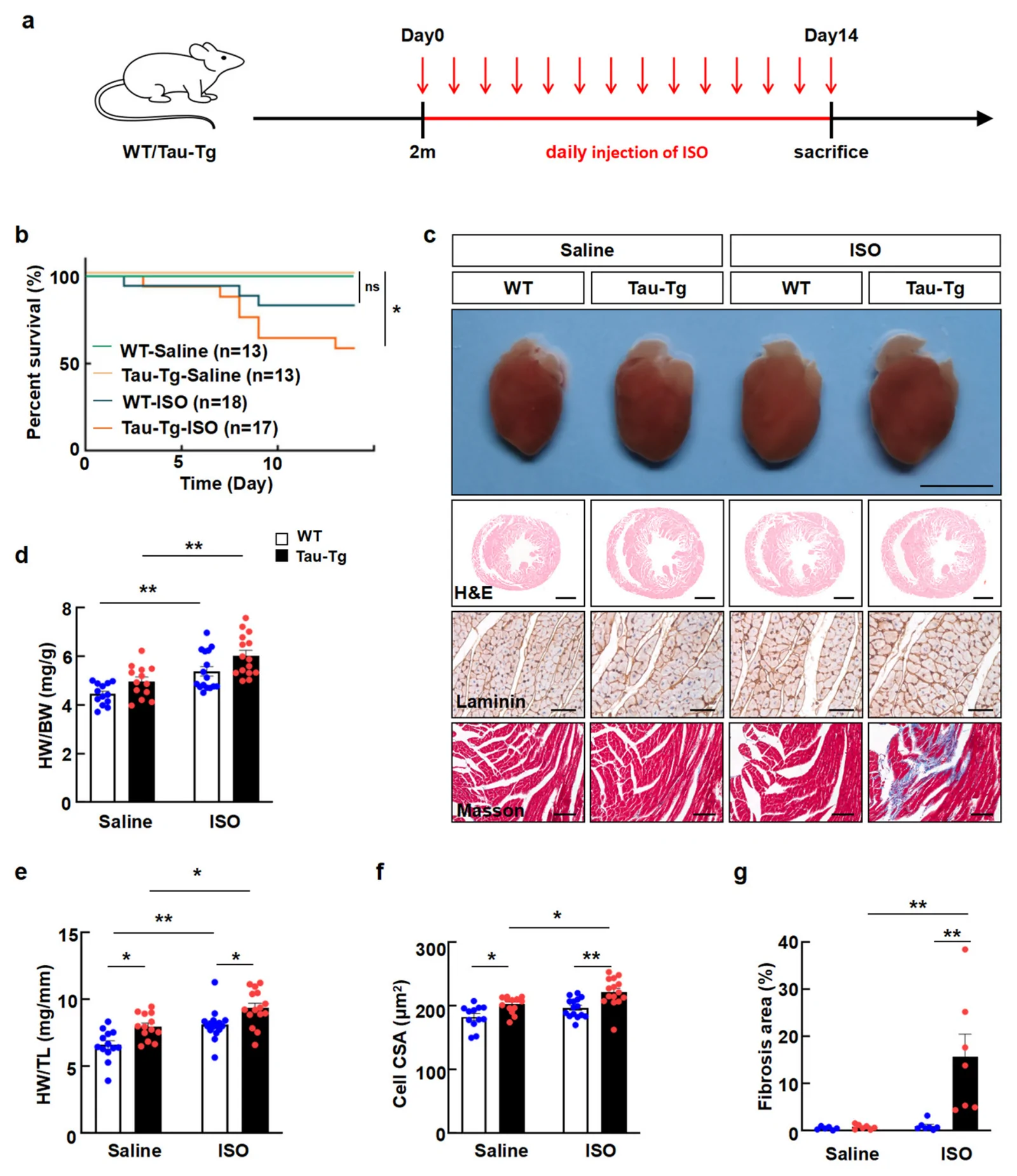
Tau overexpression aggravates ISO-induced cardiac hypertrophy. (**a**) Schematic of the experimental strategy for ISO treatment in mice (FVB/N). (**b**) Kaplan–Meier survival analysis of the indicated groups (WT-saline: n = 13; Tau-Tg-Saline: n = 13; WT-ISO: n = 18; Tau-Tg-ISO: n = 17). (**c**) Gross and histological morphology of the hearts from different groups of mice. (**Upper**): Gross morphology analysis of hearts, Scale bar, 5 mm; (**Middle**): hematoxylin and eosin (H&E)-stained cardiac sections from different groups of mice. Scale bar, 1 mm; (**Bottom**): laminin staining of heart tissue sections, Scale bar, 50 µm; (**Lower**): Masson staining of heart tissue sections, Scale bar, 100 µm. (**d**,**e**) Ratios of heart weight to body weight (HW/BW) and tibia length (HW/TL) in different groups of mice (WT-saline: n = 13; Tau-Tg-saline: n = 13; WT-ISO: n = 16; Tau-Tg-ISO: n = 15). Two-way ANOVA revealed no significant genotype × treatment interaction for HW/BW (*p* = 0.7216) or HW/TL (*p* = 0.8513), with significant main effects of both genotype and ISO treatment for both ratios. (**f**) Quantification of cell cross-sectional areas (WT-saline: n = 12; Tau-Tg-saline: n = 13; WT-ISO: n = 15; Tau-Tg-ISO: n = 15). Two-way ANOVA revealed no significant genotype × treatment interaction (*p* = 0.5553), indicating additive effects of genotype and ISO treatment on cardiomyocyte hypertrophy. (**g**) Quantification of fibrosis areas in hearts (WT-saline: n = 6; Tau-Tg-saline: n = 7; WT-ISO: n = 7; Tau-Tg-ISO: n = 7). Two-way ANOVA revealed a significant genotype × treatment interaction (*p* = 0.0085), indicating that Tau overexpression significantly exacerbates ISO-induced cardiac fibrosis. Data are presented as mean ± SEM. Statistical significance was determined by two-way ANOVA followed by Tukey’s multiple-comparisons test. * *p* < 0.05, ** *p* < 0.01.

To investigate whether cardiomyocyte-specific Tau overexpression increases susceptibility to pathological stimuli, we subjected 2-month-old Tau-Tg mice and their littermate controls to intraperitoneal injection of isoproterenol (ISO, 30 mg/kg/day) for two consecutive weeks (Figure 4a). Tau-Tg mice subjected to ISO exhibited significantly higher mortality than control littermates (Figure 4b). While ISO treatment induced myocardial hypertrophy in control mice, Tau overexpression was associated with further increases in heart weight, cardiomyocyte size, and myocardial fibrosis (Figure 4c–g). Two-way ANOVA revealed no significant genotype × treatment interaction for HW/BW (*p* = 0.7216), HW/TL (*p* = 0.8513), or cell cross-sectional area (*p* = 0.5553), indicating additive effects of Tau overexpression and ISO treatment on cardiac hypertrophy. In contrast, a significant interaction was observed for fibrosis area (*p* = 0.0085), indicating that Tau overexpression significantly exacerbated ISO-induced cardiac fibrosis. Collectively, these findings suggest that Tau overexpression differentially modulates ISO-induced cardiac remodeling, exerting additive effects on hypertrophy but synergistic effects on fibrosis, and that the exacerbated fibrotic response may contribute to the increased mortality observed in Tau-Tg mice under pathological stress.

### 2.5. Tau Overexpression Impairs Mitochondrial Function, Induces Excessive Fission, and Promotes Cardiomyocyte Apoptosis

To elucidate the mechanisms underlying Tau-induced cardiac hypertrophy, we performed RNA-seq analysis on Ad-GFP and Ad-Tau-treated NRVMs (Figure S2a). Pathway enrichment analysis of upregulated genes revealed significant activation of the hypertrophic cardiomyopathy pathway (Figure S2b), corroborating the observed hypertrophic phenotype. Notably, reactome analysis of downregulated genes revealed broad suppression of mitochondrial energy metabolism pathways, including the TCA cycle, electron transport chain, ATP synthesis, and mitochondrial translation (Figure S2c). Among these, complex I and complex IV pathways were most significantly downregulated (Figure S2d), which was corroborated by reduced mRNA levels of key subunits and assembly factors (*Ndufv2*, *Ndufb5*, *Cox17*, *Cox5b*) (Figure S2e,f).

**Figure 5:**
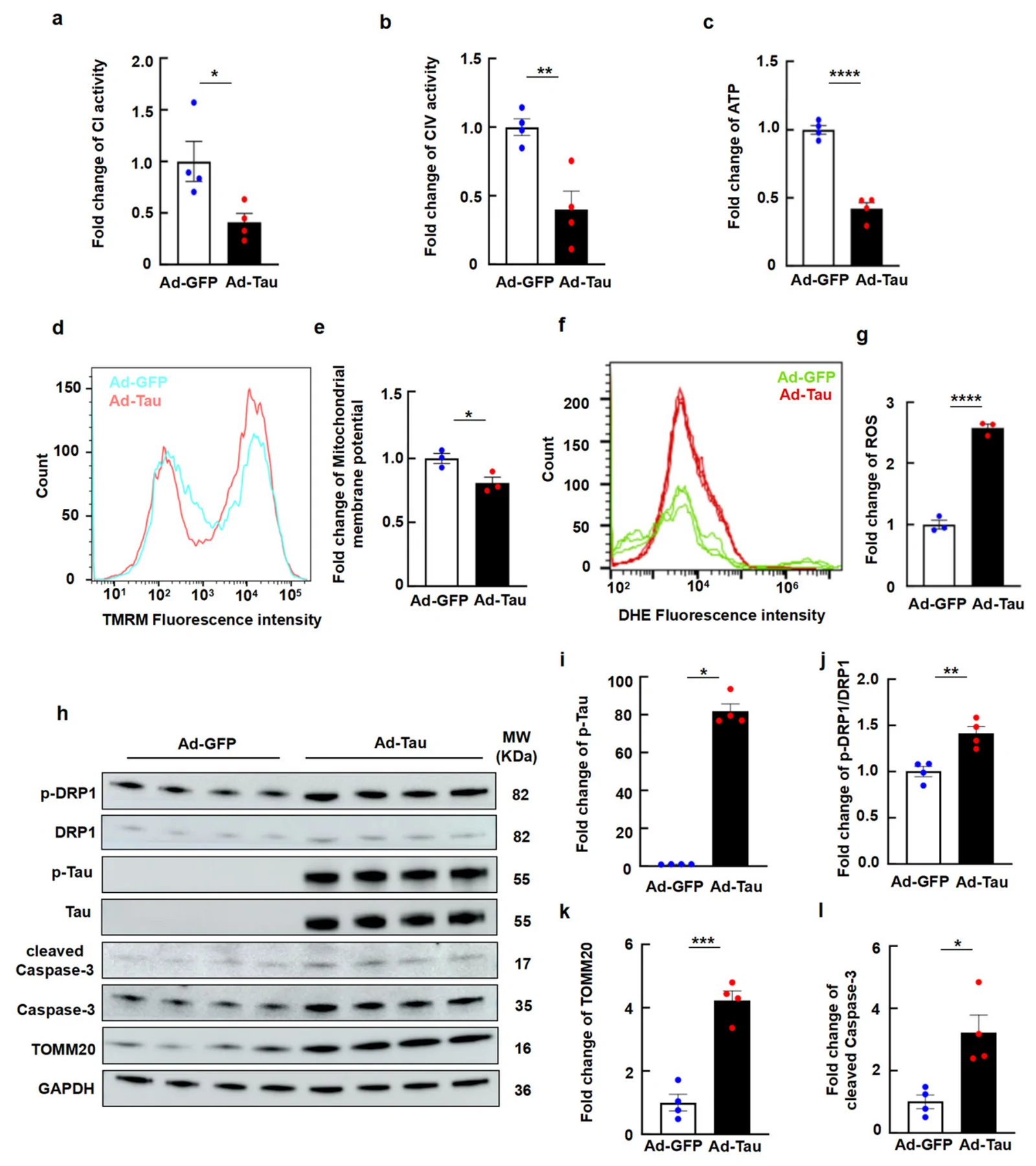
Tau overexpression impairs mitochondrial homeostasis. (**a**,**b**) Quantification of mitochondrial complex I and complex IV enzymatic activities in cardiomyocytes (n = 4 independent experiments); statistical significance was determined by a two-tailed unpaired Student’s *t*-test. (**c**) Intracellular ATP levels measured in cardiomyocytes (n = 4 independent experiments); statistical significance was determined by a two-tailed unpaired Student’s *t*-test. (**d**) Representative flow cytometry histograms of TMRM fluorescence. (**e**) Quantification of TMRM fluorescence intensity (n = 3 independent experiments); statistical significance was determined by a two-tailed unpaired Student’s *t*-test. (**f**) Quantification of DHE fluorescence intensity from imaging. (**g**) Flow cytometry-based quantification of intracellular ROS levels (n = 3 independent experiments); statistical significance was determined by a two-tailed unpaired Student’s *t*-test. (**h**) Representative Western blot of mitochondria function-related proteins in cardiomyocytes. (**i–l**) Quantification of protein expression levels normalized to the loading control (n = 4 independent experiments). For p-Tau, statistical significance was determined by the Mann–Whitney U test; for p-DRP1/DRP1,TOMM20, and cleaved-Caspase3, statistical significance was determined by a two-tailed unpaired Student’s *t*-test. Data are presented as mean ± SEM, * *p* < 0.05, ** *p* < 0.01, *** *p* < 0.001, **** *p* < 0.0001.

Functional validation confirmed that Tau overexpression significantly reduced NADH-CoQ reductase (complex I) and cytochrome c oxidase (complex IV) activities (Figure 5a,b), accompanied by marked ATP depletion (Figure 5c). Moreover, Tau-overexpressing cardiomyocytes exhibited collapsed mitochondrial membrane potential (ΔΨm) (Figure 5d,e) and elevated ROS production (Figure 5f,g). These functional deficits prompted us to examine whether Tau overexpression also disrupted mitochondrial structural dynamics. Given that mitochondrial fission is driven by Drp1, a cytosolic GTPase whose recruitment and activity are phosphorylation-dependent, and that TOMM20 serves as a mitochondrial mass marker, we next examined their expression by Western blot. Western blot analysis revealed significant upregulation of phosphorylated DRP1 at Ser616 (p-DRP1 Ser616) and TOMM20, suggesting a shift toward mitochondrial fission (Figure 5h,j,k). As excessive fission and mitochondrial dysfunction can converge to trigger apoptotic signaling, we assessed cleaved Caspase-3 expression and found it to be significantly upregulated (Figure 5h,l), confirming engagement of the apoptotic pathway.

**Figure 6:**
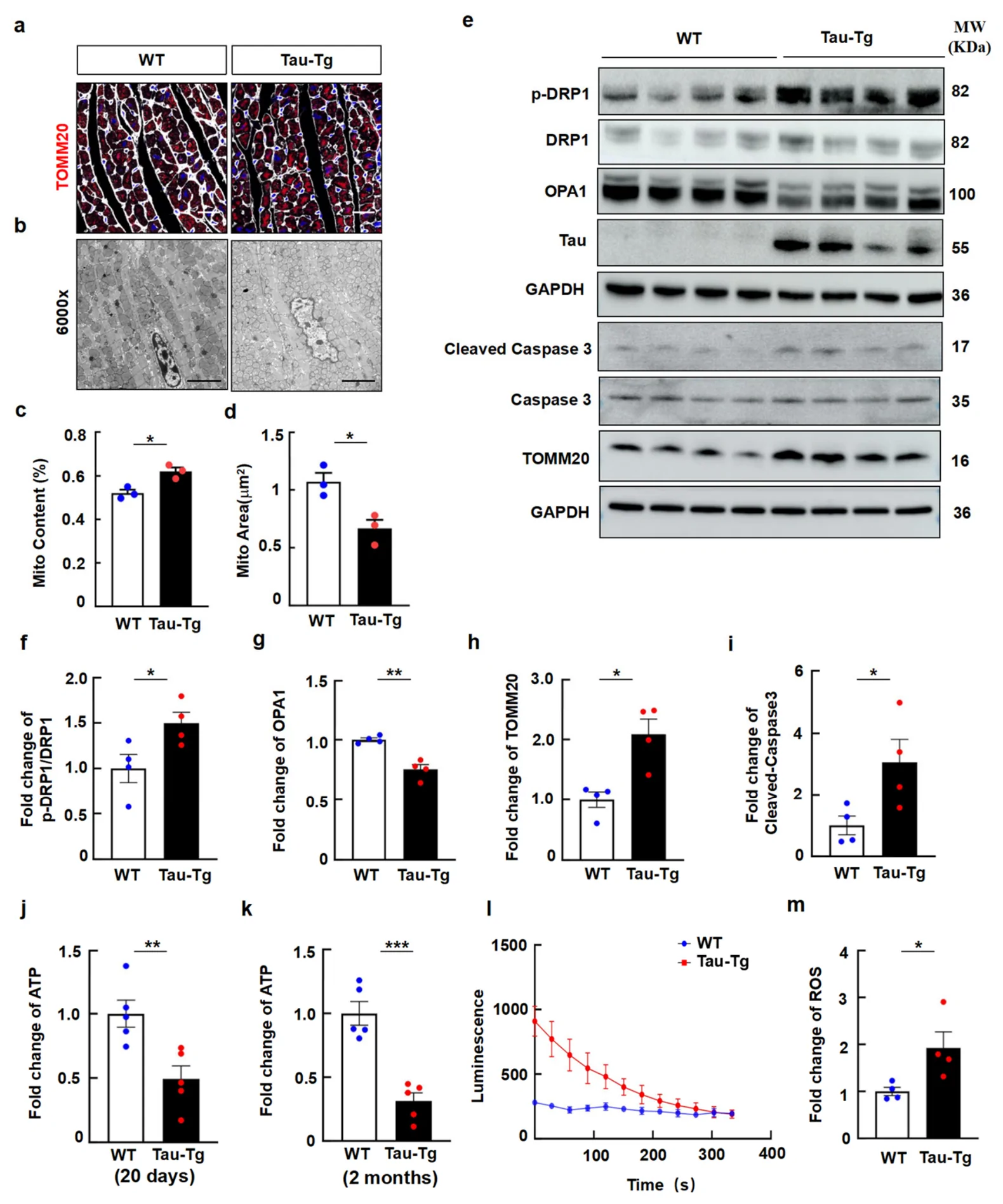
Tau overexpression impairs mitochondrial homeostasis in vivo. (**a**) Representative confocal immunofluorescence images of heart sections co-stained with anti-TOMM20 and anti-laminin antibodies. Scale bar: 20 µm. (**b**) Representative transmission electron microscopy (TEM) images of cardiac tissue ultrastructure. Scale bar: 5 μm. (**c**) Quantification of mitochondrial area as a percentage of total myocardial area (n = 3 mice per group); statistical significance was determined by two-tailed unpaired Student’s *t*-test. (**d**) Quantification of individual mitochondrial area in cardiac tissue (n = 3 mice per group); statistical significance was determined by two-tailed unpaired Student’s *t*-test. (**e**) Representative Western blots of mitochondria function-related proteins in heart tissues of mice. (**f**–**i**) Quantification of protein expression levels normalized to the loading control (n = 4 mice per group). For TOMM20, statistical significance was determined by the Mann–Whitney U test; For p-DRP1/DRP1,OPA1 and cleaved-Caspase3, statistical significance was determined by a two-tailed unpaired Student’s *t*-test. (**j**,**k**) Measurement of ATP content in cardiac tissues of 20-day-old and 2-month-old mice (n = 5 mice per group); statistical significance was determined by a two-tailed unpaired Student’s *t*-test. (**l**,**m**) Measurement of intracellular ROS levels in cardiac tissue (n = 4 mice per group); statistical significance was determined by a two-tailed unpaired Student’s *t*-test. Data are presented as mean ± SEM, * *p* < 0.05, ** *p* < 0.01, *** *p* < 0.001.

To validate these findings in vivo, we analyzed cardiac tissue from Tau transgenic mice. Immunofluorescence and transmission electron microscopy of 20-day-old transgenic hearts revealed an increased number of small mitochondrial cross-sectional profiles per area (Figure 6a–d), consistent with excessive fission. Western blot analysis further confirmed upregulation of p-DRP1 (Ser616) and TOMM20, alongside downregulation of the fusion protein OPA1 (Figure 6e–h), indicating altered mitochondrial dynamics (shifting towards fragmentation) in vivo. Consistently, cleaved Caspase-3 expression was also found to be significantly upregulated (Figure 6i) in transgenic hearts. Functionally, cardiac ATP content was already significantly reduced as early as 20 days and declined further by 2 months of age (Figure 6j,k), demonstrating progressive bioenergetic deterioration. ROS levels, assessed by the chemiluminescent probe L-012, were significantly elevated at 20 days (Figure 6l,m), confirming that oxidative stress is an early pathological event.

Collectively, these in vitro and in vivo findings demonstrate that Tau overexpression impairs mitochondrial respiratory chain function and induces imbalanced mitochondrial dynamics, leading to progressive energy deficiency, oxidative stress, and cardiomyocyte apoptosis.

### 2.6. Pharmacological Inhibition of Mitochondrial Fission Rescues Tau-Induced Mitochondrial Dysfunction and Cardiomyocyte Hypertrophy

**Figure 7:**
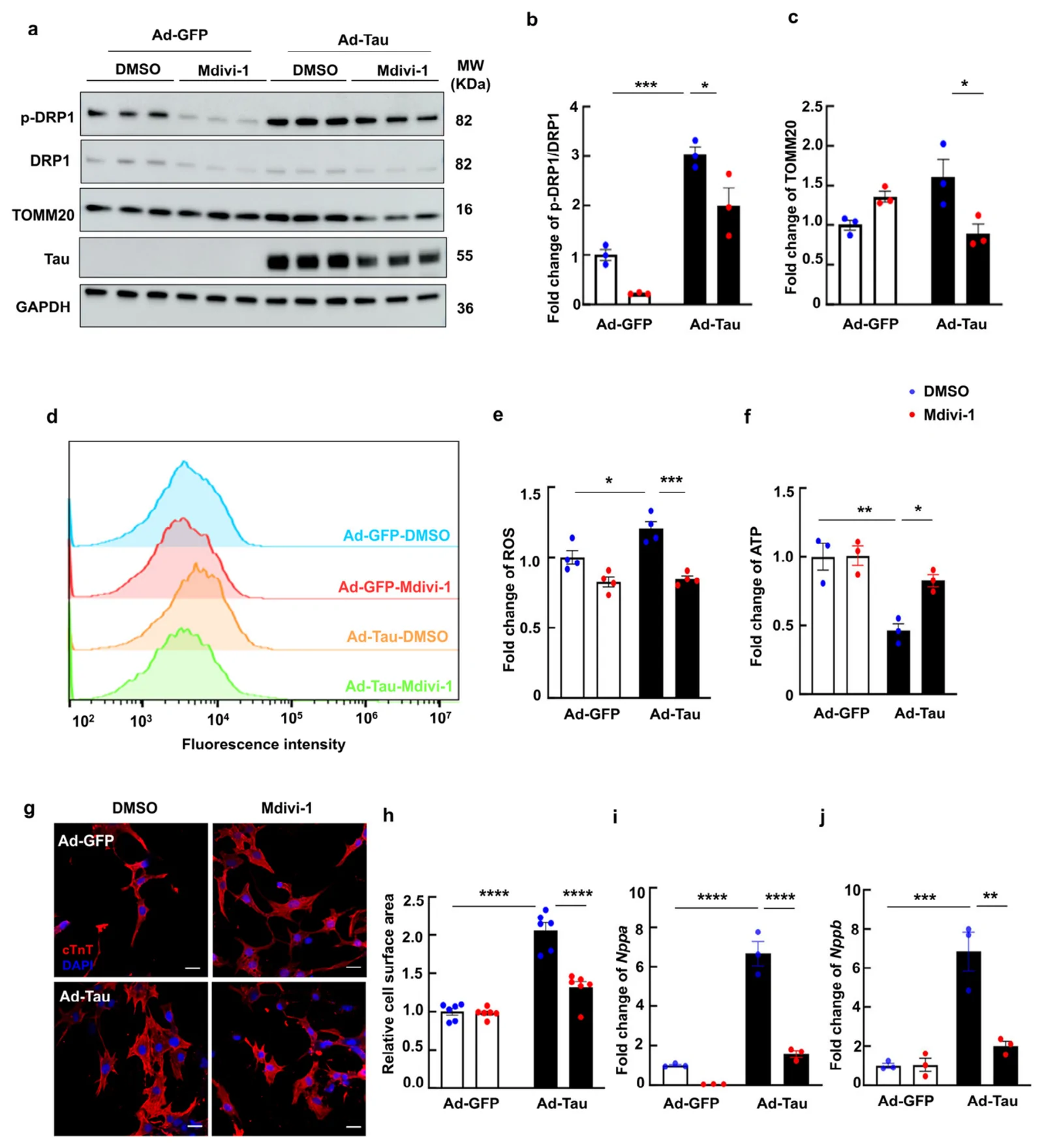
Pharmacological inhibition of mitochondrial fission by Mdivi-1 rescues Tau-induced mitochondrial dysfunction and attenuates cardiomyocyte hypertrophy. (**a**) Representative Western blot of mitochondria-related proteins in cardiomyocytes from the indicated treatment groups. (**b**,**c**) Quantification of protein expression levels normalized to the loading control (n = 3 independent experiments). For genotype × treatment interaction, interaction *p*-values: p-DRP1/drp1, *p* = 0.5486; TOMM20, *p* = 0.0040. (**d**,**e**) Flow cytometry-based assessment of intracellular ROS levels in cardiomyocytes across treatment groups (n = 4 independent experiments), interaction *p* = 0.0441. (**f**) Quantification of relative intracellular ATP content in cardiomyocytes (n = 3 independent experiments). interaction *p* = 0.0324. (**g**) Representative immunofluorescence images of NRVMs stained for cTnT (red) with DAPI counterstaining (blue). Scale bar: 20 µm. (**h**) Quantification of relative cell surface area of cardiomyocytes (n = 6 independent experiments), interaction *p* < 0.0001. (**i**,**j**) Real-time PCR analysis of *Nppa* and *Nppb* mRNA expression in cardiomyocytes (n = 3 independent experiments). Interaction *p*-values: *Nppa*, *p* = 0.0002; *Nppb*, *p* = 0.0021. Data are presented as mean ± SEM. Statistical significance was determined by two-way ANOVA followed by Tukey’s multiple-comparisons test, * *p* < 0.05, ** *p* < 0.01, *** *p* < 0.001, **** *p* < 0.0001.

To pharmacologically modulate mitochondrial dynamics, we treated Tau-overexpressing primary cardiomyocytes with Mdivi-1, a widely used pharmacological inhibitor of mitochondrial fission that blocks excessive mitochondrial fission and restores mitochondrial dynamics balance [15]. Mdivi-1 treatment significantly reduced p-DRP1 (Ser616) phosphorylation and TOMM20 expression in Tau-overexpressing cardiomyocytes (Figure 7a–c), consistent with reduced mitochondrial fragmentation. Two-way ANOVA revealed no significant genotype × treatment interaction for p-DRP1 (*p* = 0.5486), indicating that Mdivi-1 reduced DRP1 phosphorylation irrespective of Tau overexpression. Mdivi-1 also significantly reduced TOMM20 expression with a significant interaction (*p* = 0.0040), suggesting that the effect on mitochondrial outer membrane protein levels was dependent on the Tau overexpression background. Functionally, Mdivi-1 did not significantly alter these parameters under basal conditions, while markedly attenuating the Tau-overexpression-induced elevation of intracellular ROS, as assessed by DHE staining and flow cytometry (Figure 7d,e; interaction *p* = 0.0441), and restoring intracellular ATP content (Figure 7f; interaction *p* = 0.0324). These results suggest that pharmacological inhibition of mitochondrial fission ameliorates both the oxidative stress and bioenergetic failure caused by Tau overexpression.

Having shown that Mdivi-1 attenuated mitochondrial dysfunction in cultured primary cardiomyocytes, we next examined its effect on the hypertrophic growth phenotype in the same in vitro system. Quantification of relative cell surface area by immunofluorescence staining for cTnT in primary cardiomyocytes demonstrated that Mdivi-1 treatment significantly attenuated Tau overexpression-induced cardiomyocyte hypertrophy (Figure 7g,h; two-way ANOVA: genotype × treatment interaction *p* < 0.0001). Real-time PCR analysis on these cells revealed that Tau overexpression significantly upregulated the mRNA expression of cardiac hypertrophy marker genes *Nppa* and *Nppb*, both of which were significantly suppressed by Mdivi-1 treatment (Figure 7i,j; interaction *p*-values: *Nppa*, *p* = 0.0002; *Nppb*, *p* = 0.0021). Taken together, these findings suggest that inhibiting excessive mitochondrial fission with Mdivi-1 attenuates Tau overexpression-induced mitochondrial dysfunction and cardiomyocyte hypertrophic growth, consistent with a contribution of dysregulated mitochondrial dynamics to Tau-associated cardiac remodeling.

## 3. Discussion

Converging evidence suggests a mechanistic connection between neurodegenerative proteinopathies and cardiac disease [16]. Beyond its well-characterized brain pathology, amyloid beta has also been detected in the human heart and associated with diastolic dysfunction [17,18]. Clinically, serum pTau-181 levels rise progressively in patients with chronic heart failure over a three-year follow-up period [19,20]. Furthermore, a high-molecular-weight (HMW) Tau isoform is demonstrated to be expressed in both normal and diseased human hearts, with hyperphosphorylated Tau existing in aggregated form in heart failure and Alzheimer’s patients [14]. Despite these associative findings, a causal role for Tau in cardiac pathology has remained unproven. Here, we provide genetic evidence that ~55 kDa Tau-immunoreactive species acts as a cardiomyocyte-intrinsic factor sufficient to promote pathological remodeling. We show that this species is stress-inducible, capable of initiating hypertrophy at baseline, and is associated with an exacerbated response to ISO. To our knowledge, this is the first demonstration that Tau actively contributes to cardiac hypertrophy and remodeling, suggesting a possible functional link between Tau pathology in the nervous system and its pathogenic role in the heart.

Mechanistically, our findings converge on Tau-induced mitochondrial dyshomeostasis as the central pathogenic event in cardiomyocytes. Prior studies in neurons have established that Tau perturbs mitochondrial dynamics and bioenergetics by altering fusion/fission balance, promoting perinuclear clustering, and suppressing complex I activity [21,22,23]. Extending these observations to the heart, our transcriptomic and functional analyses show that Tau overexpression elicits a mitochondrial phenotype characterized by broad downregulation of respiratory chain components (particularly complex I and IV subunits), increased DRP1 phosphorylation associated with mitochondrial fragmentation, and consequent bioenergetic failure, oxidative stress, and apoptotic signaling. These findings suggest that the mitochondrial perturbations previously described in the nervous system may also occur in cardiomyocytes upon Tau overexpression, raising the possibility that Tau-mediated mitochondrial disruption represents a shared pathogenic mechanism linking neurodegenerative and cardiovascular diseases. Pharmacological intervention further supports the association of Tau overexpression-induced mitochondrial dysfunction with cardiac hypertrophy. Treatment of Tau-overexpressing cardiomyocytes with Mdivi-1 partially normalized mitochondrial function and attenuated the hypertrophic phenotype. While off-target effects of Mdivi-1 [24,25] warrant caution in interpreting mechanistic specificity, the observed partial rescue is consistent with a contribution of dysregulated mitochondrial fission to Tau-associated hypertrophic remodeling. These findings suggest that correcting mitochondrial dysfunction may warrant further exploration as a potential therapeutic strategy for Tau-related cardiac disease.

Several limitations should be acknowledged. First, while our data establish a functional link between Tau and mitochondrial fission, the precise molecular interface through which Tau engages the DRP1 phosphorylation machinery in cardiomyocytes remains undefined. Whether Tau directly interacts with DRP1, as suggested in neuronal systems [26], or acts through upstream kinases/phosphatases specific to the cardiac context requires further investigation. Second, given the limited selectivity of Mdivi-1 as a DRP1 inhibitor, off-target effects cannot be formally excluded, and genetic loss-of-function models (e.g., cardiomyocyte-specific Drp1 knockout) or peptide-based modulators will be necessary to more definitively assess the contribution of DRP1-mediated fission to Tau-associated cardiac remodeling. Third, although our gain-of-function data establish that Tau overexpression is sufficient to promote pathological remodeling, the supraphysiological expression levels in these models may not fully recapitulate the modest endogenous changes seen in acquired cardiac disease; thus, whether endogenous Tau is necessary for stress-induced cardiac pathology warrants future investigation. Finally, despite overall similarity between human and murine Tau, species-specific splicing differences result in distinct isoform compositions; the ~55 kDa Tau species detected in our mouse model therefore cannot be unequivocally assigned to a specific human isoform. Accordingly, the translational relevance of these findings requires validation in human iPSC-derived cardiomyocytes and well-characterized patient cohorts, which will also help determine whether circulating or cardiac Tau species may serve as biomarkers for heart failure risk stratification.

In summary, this study identifies the ~55 kDa Tau species as a stress-associated factor that links pathological stress to mitochondrial fission and cardiac remodeling. By providing evidence for an association between Tau dysregulation and mitochondrial dysfunction in the heart, our findings offer a mechanistic framework that may help explain the elevated cardiovascular morbidity observed in patients with neurodegenerative tauopathies. Importantly, the convergence of Tau-associated mitochondrial pathology across the brain and heart raises the possibility that therapeutic strategies targeting Tau aggregation, Tau–mitochondria interaction, or fission machinery may have relevance for both neurological and cardiac outcomes—a prospect that warrants further investigation in preclinical models of comorbid disease.

## 4. Materials and Methods

### 4.1. Animal Models

Neonatal SD rats, 1–3 days after birth, were purchased from Beijing Vital River Laboratory Animal Technology Co., Ltd. Cardiomyocyte-specific Tau overexpression transgenic mice were generated, housed, and bred in our laboratory. Briefly, Tau-Tg mice were generated by microinjection of a transgene construct containing the Tau-encoding sequence (NCBI Reference Sequence NM_001038609.3; CDS spanning nucleotides 143–1435, 1293 bp, encoding 430 amino acids with a predicted molecular mass of ≈47.3 kDa) under the control of the murine α-myosin heavy chain (α-MHC) promoter into fertilized FVB oocytes. Founders were identified by PCR genotyping of tail genomic DNA. Age- and sex-matched littermates lacking the transgene served as controls. The identical CDS fragment was also used to construct an adenoviral vector (Ad-Tau) for supplementary experiments.

Two conceptually distinct isoproterenol (ISO) experiments were performed. Experiment 1 (endogenous Tau response): 2-month-old male C57BL/6J mice received daily intraperitoneal injections of ISO (30 mg/kg/day, Sigma-Aldrich, St. Louis, MO, USA, I5627) dissolved in sterile saline for 7 or 14 consecutive days; control animals received equivalent volumes of saline vehicle. Experiment 2 (Tau-Tg vs. littermates): 2-month-old male Tau-Tg mice (strain background: FVB/N) and their wild-type (WT) littermates received daily intraperitoneal injections of ISO (30 mg/kg/day) or saline vehicle for 14 consecutive days. All animals were maintained, and experimental procedures were conducted in a specific pathogen-free (SPF) facility in accordance with protocols approved by the Institutional Animal Care and Use Committee.

For HW/BW and HW/TL ratio calculations, body weight (BW) was first measured using an analytical balance, after which mice were euthanized at the indicated time points. Hearts were rapidly excised, rinsed in ice-cold PBS to remove residual blood, blotted dry, and weighed to obtain heart weight (HW). Tibia length (TL) was measured from the tibial plateau to the medial malleolus using a vernier caliper after careful removal of the surrounding soft tissues. HW/BW and HW/TL ratios were then calculated.

### 4.2. Cell Culture and Transfection

In brief, ventricles were cut into small pieces and digested using a Neonatal Heart Dissociation Kit (Miltenyi Biotec, Bergisch Gladbach, Germany, 130-098-373) on a gentleMACS Octo Dissociator (Miltenyi Biotec, Bergisch Gladbach, Germany, 130-096-427) according to the manufacturer’s instructions. Digestion was terminated by adding three volumes of DMEM/F12 medium (Cytiva, Marlborough, MA, USA, SH30023.01) containing 15% fetal bovine serum (BioIndustries, Kibbutz Beit Haemek, Israel, 04-001-1ACS). The cell suspension was filtered through a 100 μm cell strainer (Biologix, Jinan, China, 15-1100) and centrifuged at 1000 rpm for 10 min. The supernatant was discarded, and the cell pellet was resuspended gently in medium and centrifuged again at 1000 rpm for 6 min. After discarding the supernatant, cells were resuspended in DMEM/F12 supplemented with 15% fetal bovine serum and 100 μM 5-bromo-2’-deoxyuridine (Sigma, St. Louis, MO, USA, B9285) and plated onto 10 cm culture dishes for 2 h at 37 °C to remove fibroblasts by differential adhesion. Non-adherent cells in the supernatant were collected and seeded onto 1% gelatin-coated culture dishes. After 48 h of culture, the culture was replaced with serum-free DMEM/F12 medium, and cells were cultured for an additional 24 h. Subsequently, the medium was replaced with serum-free DMEM/F12 containing Ad-GFP or Ad-Tau, and cells were cultured for 48 h. Mdivi-1 (Sigma, St. Louis, MO, USA, M0199, 30 μM) was dissolved in DMSO. For all in vitro experiments, biological replicates correspond to independent cardiomyocyte isolations performed on different days. For each isolation, cardiomyocytes were pooled from multiple pups from the same litter and split into the different treatment groups. While hundreds of individual cells were measured per group, these were treated as technical replicates, and statistical analyses were performed on the averaged values of the independent isolations.

### 4.3. Histological and Immunofluorescence Assay

Hearts were fixed in 4% PFA overnight at 4 °C. Following dehydration, hearts were embedded in paraffin and sectioned at 5 μm thickness. Sections were deparaffinized by baking followed by rehydration. Sections were stained with hematoxylin and eosin (H&E) to examine myocardial morphology and with Masson’s trichrome stain (Solarbio Life Sciences, Beijing, China, G1346) to assess fibrosis. For immunofluorescence analysis of heart tissue, sections underwent heat-mediated antigen retrieval in sodium citrate buffer (pH 6.0), followed by treatment with 3% hydrogen peroxide for 15 min and blocking with goat serum for 40 min at 37 °C. Sections were then incubated with primary antibodies overnight at 4 °C in the dark, followed by incubation with HRP-conjugated secondary antibody (ZSGB-BIO, Beijing, China, PV-6001) at 37 °C for 40 min with tyramide signal amplification (TSA) fluorescent detection (Histova Biotechnology, Beijing, China, NECC7100). Finally, sections were counterstained with Hoechst and imaged using a Zeiss LSM 880 confocal laser scanning microscope (Carl Zeiss, Oberkochen, Germany).

For quantification of fibrosis, Masson’s trichrome-stained sections were imaged using a light microscope at 20× magnification, and three randomly selected fields per section were analyzed. Fibrosis areas were quantified using ImageJ software (version 1.54g, National Institutes of Health, Bethesda, MD, USA) by color thresholding to select blue-stained fibrotic regions and red-stained cardiomyocyte regions, respectively. Fibrosis was expressed as the percentage of blue-stained area relative to the total red-stained cardiomyocyte area within a single field, and values from the three fields were averaged per section. All quantifications were performed by an investigator blinded to genotype.

For cardiomyocyte cross-sectional area measurement, heart sections stained with laminin (Sigma-Aldrich, St. Louis, MO, USA, L9393, 1:25) were imaged using a confocal microscope (Zeiss LSM 880, Carl Zeiss, Oberkochen, Germany). For each animal, at least 200 cardiomyocytes were randomly selected and manually traced along the laminin-stained cell border using ImageJ software (version 1.54g, National Institutes of Health, Bethesda, MD, USA). The cross-sectional area of each myocyte was calculated, and values were averaged per animal for statistical analysis. All measurements were performed by an investigator blinded to genotype.

For immunofluorescence analysis of primary neonatal cardiomyocytes, cells were fixed with 4% formaldehyde for 30 min at 4 °C and permeabilized with 0.5% Triton X-100 in PBS for 15 min at room temperature. Cells were then incubated with primary antibody against cardiac Troponin T (cTnT, 15513-1-AP, Proteintech, Rosemont, IL, USA, 1:200) overnight at 4 °C, followed by incubation with fluorescence-conjugated secondary antibody. Nuclei were counterstained with DAPI for 10 min. Images were acquired using a confocal microscope (Carl Zeiss, Oberkochen, Germany, LSM 880). For cardiomyocyte surface area measurement, at least 200 randomly selected cells per independent experiment were manually traced along the cTnT-stained cell border using ImageJ software (version 1.54g, National Institutes of Health, Bethesda, MD, USA). The surface area of each cell was calculated, and values were averaged per independent experiment for statistical analysis. All measurements were performed by an investigator blinded to treatment conditions.

### 4.4. Quantitative Real-Time PCR

Total RNA was extracted using TRIzol reagent (Invitrogen, Carlsbad, CA, USA, 15596026), and cDNA was synthesized from 2000 ng of total RNA using a reverse transcription kit (TOYOBO, Osaka, Japan, FSQ-201). Real-time quantitative PCR (qPCR) was performed using SYBR Green Real-time PCR Master Mix (TOYOBO, Osaka, Japan, QPK-201) on a 7500 Fast Real-Time PCR System (Applied Biosystems, Foster City, CA, USA). The thermocycler conditions were as follows: initial denaturation at 95 °C for 1 min; followed by 40 cycles of denaturation at 95 °C for 15 s, annealing at 60 °C for 15 s, and extension at 72 °C for 45 s; followed by melt curve analysis from 60 °C to 95 °C at 0.1 °C/s. Relative mRNA expression was calculated using the 2^−ΔΔCt^ method. Gene expression was normalized to Gapdh and expressed relative to controls. Primer sequences are listed in Supplementary Table S2.

### 4.5. RNA Sequencing

Total RNA was extracted from primary rat cardiomyocytes infected with either Ad-GFP or Ad-Tau, using TRIzol reagent (Invitrogen, Carlsbad, CA, USA, 15596026). Three independent biological replicates were prepared per group. RNA integrity was assessed using a Fragment Analyzer (Agilent, Santa Clara, CA, USA), confirming high quality for all six samples, as indicated by RIN values of 9.0–9.7, 28S/18S ratios of 1.5–3.2, and total RNA yields of 0.43–0.56 µg. Sequencing libraries were constructed and sequenced on the DNBSEQ platform (BGI, Shenzhen, China), generating an average of 6.65 Gb of 150 bp paired-end reads per sample. Raw data quality metrics showed average Q20 and Q30 scores of >97.9% and >93.2%, respectively. Raw reads were filtered using SOAPnuke (v1.5.6) to remove adapters, poly-A sequences, reads with >20% low-quality bases (Q ≤ 15), or reads with >0.1% unknown bases (N). The resulting clean reads were used for all downstream analyses performed on the Dr. Tom Multiomics Data Mining System (https://biosys.bgi.com).

Clean reads were aligned to the Rattus norvegicus reference genome (Rnor_6.0, NCBI accession: GCF_000001895.5) using HISAT2 (v2.2.1). The total genome mapping rates were 60.77%, 63.13%, and 64.58% for the control samples (Control 1 to Control 3) and 93.51%, 94.02%, and 93.32% for the Tau samples (Tau 1 to Tau 3). The corresponding unique mapping rates were 57.09%, 57.97%, and 60.78% for the control group, and 86.91%, 87.31%, and 86.43% for the Tau group. The observed group-dependent difference in mapping rates is likely attributable to the presence of adenoviral transgene-derived reads. Specifically, in the Tau-overexpressing group, reads originating from the mouse-derived *Mapt* transgene share high homology (~90–95%) with the endogenous rat *Mapt* gene and can therefore align to the rat *Mapt* locus, contributing to the higher mapping rates. In contrast, GFP-derived reads in the control group and reads from the viral vector backbone in both groups lack a rat genomic match and remain unmapped. All such non-aligning reads are excluded from the gene-level count matrix.

For transcript quantification, clean reads were also aligned to a comprehensive gene set (including known and novel, coding and noncoding transcripts) using Bowtie2 (v2.3.4.3), and expression levels were calculated using RSEM (v1.2.28). To ensure that biological signal dominated over technical variation, Principal Component Analysis (PCA) was performed on the expression profiles. This analysis confirmed a clear separation by treatment group rather than by mapping efficiency, and DESeq2’s internal size-factor normalization effectively corrected for differences in effective library size. For differential expression analysis, RSEM-generated expected counts were rounded to integers and used as input for DESeq2 (v1.40.2). Genes with a raw count ≥10 in at least two samples were retained for analysis, resulting in 12,435 genes tested. Differentially expressed genes (DEGs) were defined by a fold change >1.5 and a false discovery rate (FDR)-adjusted *p*-value (q value) < 0.05. FPKM and TPM values were calculated by RSEM solely for data visualization purposes. A heatmap was generated using pheatmap (v1.0.10). Gene Ontology (GO) enrichment analysis of the annotated DEGs was performed using Phyper, which is based on the hypergeometric test. The significance of enriched terms and pathways was corrected for multiple testing using a Q value threshold of ≤0.05.

### 4.6. Western Blot

Proteins were extracted from whole heart lysate homogenized in RIPA lysis buffer (Applygen, Beijing, China, C1053) with protease and phosphatase inhibitors, and quantified using Pierce BCA Protein Assay Reagent (Thermo Fisher Scientific, Waltham, MA, USA, 23225) as described [27].

Thirty micrograms of protein were separated by electrophoresis on a 12% Bis-Tris precast polyacrylamide gel (36261ES10, Yeasen Biotechnology, Shanghai, China) at 100 V constant voltage for 1 h in Tris-Mops running buffer (36271ES05, Yeasen Biotechnology, Shanghai, China). Proteins were then transferred onto a PVDF membrane (Immobilon^®^-P, Merck Millipore, Darmstadt, Germany, IPVH00010, 0.45 μm pore size) using a rapid transfer buffer (Servicebio, Wuhan, China, G2148-1L) at 300 mA constant current for 30 min. Membranes were blocked in 10% milk in PBST for 1 hour at room temperature, and incubated with primary antibodies at 4 °C overnight. Immunoreactive bands were detected using Enlight™ Western Blot Substrate (Engreen Biosystem, Beijing, China, 29100). The primary antibodies used for Western blotting are listed in Supplementary Table S3, along with their sources, catalog numbers, and dilution ratios.

### 4.7. ATP Measurement

ATP levels in NRVMs and tissue were measured using the Enhanced ATP Assay Kit (Beyotime, Shanghai, China, S0027) according to the manufacturer’s instructions. The assay is based on firefly luciferase-catalyzed conversion of luciferin to oxyluciferin, which produces luminescence proportional to ATP concentration. ATP detection working solution was prepared by diluting the ATP detection reagent with ATP detection reagent diluent at a 1:4 ratio. For each measurement, 100 μL of ATP detection working solution was added to each well and incubated at room temperature for 3–5 min to deplete background ATP. Then, 20 μL of samples or ATP standards (prepared by serial dilution of ATP standard solution in lysis buffer, ranging from 0.01 to 10 μM) were added to each well, and luminescence was immediately recorded using a chemiluminescence imaging analyzer (ImageQuant LAS 4000, GE Healthcare, Chicago, IL, USA) with a 2 s delay and a 10 s integration time. ATP concentrations were calculated from the standard curve of each independent experiment. ATP levels were normalized to the protein content of each sample, as determined by BCA assay (Thermo Fisher Scientific, Waltham, MA, USA, 23225).

### 4.8. Measurement of Mitochondrial Respiratory Chain Complex Activities

The activity of OXPHOS complexes was measured using enzyme activity assay kits according to the manufacturer’s protocols. Briefly, mitochondria were extracted from cultured NRVMs using the lysis buffer provided in the kit. Mitochondria-containing pellets were resuspended in lysis buffer and subjected to ultrasonic disruption (200 W, 5 s on/10 s off, 15 cycles) on ice to release the enzymes. Mitochondrial complex I activity was assessed by measuring NADH-coenzyme Q reductase activity (Solarbio, Beijing, China, BC0515) at 37 °C. The assay mixture contained 10 μL of sample, 154 μL of reagent I, 20 μL of working solution, and 16 μL of reagent IV in a final volume of 200 μL. The decrease in absorbance at 340 nm (ε = 6.22 × 10^3^ L/mol/cm) was monitored at 10 s and 1 min 10 s after initiation; background readings were subtracted using a blank control (distilled water instead of sample). Complex I activity was calculated as: Complex I activity (U/mg prot) = 5359.05 × ΔA ÷ Cpr, where ΔA is the decrease in absorbance at 340 nm (A_10_s − A_1min10_s) after subtracting the blank, and Cpr is the protein concentration (mg/mL) of the sample. One unit of complex I activity was defined as the consumption of 1 nmol of NADH per minute per mg of protein. Mitochondrial complex IV activity was assessed by measuring cytochrome c oxidase activity (Solarbio, Beijing, China, BC0945) at 37 °C. The assay mixture contained 10 μL of sample and 200 μL of working solution in a final volume of 210 μL. The decrease in absorbance at 550 nm (ε = 1.91 × 10^4^ L/mol/cm) was monitored at 10 s and 1 min after initiation, and background readings were subtracted using a blank control (distilled water instead of sample). Complex IV activity was calculated as: Complex IV activity (U/mg prot) = 1832 × ΔA ÷ Cpr, where ΔA is the decrease in absorbance at 550 nm (A_10_s − A_1min_) after subtracting the blank, and Cpr is the protein concentration (mg/mL) of the sample. One unit of complex IV activity was defined as the oxidation of 1 nmol of reduced cytochrome c per minute per mg of protein. All absorbance measurements were performed using a microplate reader (SpectraMax Plus, Molecular Devices, San Jose, CA, USA) equipped with UV-transparent 96-well plates (for complex I) or 96-well plates (for complex IV). Enzyme activities were normalized to the protein content of each sample, as determined by a BCA assay (Thermo Fisher Scientific, Waltham, MA, USA, 23225).

### 4.9. Flow Cytometry Analysis of Mitochondrial Membrane Potential and ROS

Following treatment, 1 × 10^6^ NRVMs were collected and stained with specific fluorescent dyes according to the manufacturer’s protocols. For mitochondrial membrane potential (ΔΨm) measurement, cells were incubated with TMRM (0.2 μM, Thermo Fisher Scientific, Waltham, MA, USA, T669) for 20 min at 37 °C in the dark. For intracellular ROS detection, cells were incubated with dihydroethidium (DHE, 5 μM, Beyotime, Shanghai, China, S0063) for 30 min at 37 °C in the dark. Following staining, cells were washed three times with PBS and centrifuged at 800 rpm for 5 min at 25 °C. Supernatants were discarded, and cell pellets were resuspended in 200 μL of assay buffer. Fluorescence was acquired using the PE channel for both TMRM and DHE on a CytoFLEX flow cytometer (Beckman Coulter, Brea, CA, USA), and 10,000 events were collected per sample. Finally, the labeled cells were analyzed by FlowJo v10.10.0 CL software (BD Biosciences, Ashland, OR, USA). For all flow cytometry analyses, biological replicates correspond to independent cardiomyocyte isolations performed on different days. For each isolation, cells from the same isolation were split into the different treatment groups. While thousands of individual cells were analyzed per group, these were treated as technical replicates, and statistical analyses were performed on the averaged values of the independent isolations. Fluorescence intensities were normalized to the mean value of the control group for each independent experiment.

### 4.10. ROS Detection in Cardiac Tissue

Heart tissues from 20-day-old mice were homogenized in ice-cold HEPES buffer (20 mM, pH 7.4) containing 0.1% Triton X-100. Homogenates were centrifuged at 12,000× *g* for 15 min at 4 °C, and supernatants were collected. Protein concentrations were determined by BCA assay. Equivalent amounts of protein (5 μg) were mixed with L-012 chemiluminescent probe (MedChemExpress (MCE), Monmouth Junction, NJ, USA, HY-108537) at a final concentration of 200 μM in a 96-well white plate. Luminescence signals were immediately recorded every 2 min for 30 min using a chemiluminescence imaging analyzer (ImageQuant LAS 4000, GE Healthcare, Chicago, IL, USA). ROS levels were expressed as relative light units (RLU) per mg protein.

### 4.11. Cardiac Ultrasound Imaging

Echocardiography was conducted with a Vevo 2100 ultrasound system (VisualSonics, Toronto, ON, Canada) equipped with a 30 MHz linear array transducer. Assessments were performed on independent, age-matched cohorts of mice at 2, 10, and 17 months, rather than as a longitudinal follow-up of the same animals. The mice were anesthetized with 3% isoflurane, the hair on the chest wall and upper abdomen was removed with depilation cream, and the animals were placed supine on the ultrasound platform and fixed. The parasternal long-axis view of the left ventricular inflow tract was obtained, and the probe was slightly angled toward the left chest to clearly visualize the left atrium and ventricular inflow tract. The width and length of the left ventricle were maximized to obtain B-mode ultrasound images, and then M-mode ultrasound images were acquired at the level of the mitral chordae tendineae. Left ventricular (LV) internal dimensions at end-diastole (LVIDd) and end-systole (LVIDs), LV posterior wall thickness at end-diastole (LVPWd) and end-systole (LVPWs), and LV anterior wall thickness at end-diastole (LVAWd) and end-systole (LVAWs) were measured from M-mode tracings. LV ejection fraction (LVEF) and fractional shortening (FS) were calculated using VevoLab software (v3.2.6, FUJIFILM VisualSonics, Toronto, ON, Canada). All measurements were averaged from at least three consecutive cardiac cycles by an investigator blinded to genotype.

### 4.12. Transmission Electron Microscopy (TEM)

For transmission electron microscopy (TEM) analysis, heart tissues from mice were dissected and cut into 1 mm^3^ pieces. Samples were fixed in 3% glutaraldehyde in 0.075 M phosphate-buffered saline (PBS, pH 7.4) at 4 °C for 2 h, followed by rinsing in 0.075 M PBS containing 0.19 M sucrose at 4 °C. Samples were then post-fixed in 1% osmium tetroxide in 0.24 M PBS (pH 7.4) at 4 °C for 2 h and rinsed again in 0.075 M PBS with 0.19 M sucrose at 4 °C for 15 min. Dehydration was performed through a graded ethanol series (50%, 70%, 90%, each for 5–10 min at 4 °C), followed by 90% ethanol:90% acetone (1:1) for 5–10 min at 4 °C, 90% acetone for 5–10 min at 4 °C, and finally 100% acetone twice (first for 10–15 min at 4 °C, then for 10–15 min at room temperature). Samples were infiltrated with a 1:1 mixture of 100% acetone and embedding resin (Epon 812, Electron Microscopy Sciences, Hatfield, PA, USA) for 30 min at room temperature, followed by pure resin infiltration overnight. Polymerization was performed at 35 °C for 12 h, 45 °C for 12 h, and 60 °C for 24 h. Ultrathin sections (70 nm) were cut using an ultramicrotome (Leica EM UC7, Leica Microsystems, Wetzlar, Germany). For mitochondrial morphology assessment, sections were stained with uranyl acetate for 10 min and lead citrate for 10 min, and imaged using a transmission electron microscope (Hitachi H-7650, Hitachi, Tokyo, Japan) at 12,000× magnification. Mitochondria were identified based on their characteristic double-membrane structures and cristae. For each animal, five randomly selected fields were analyzed, and at least 50 mitochondria per animal were manually traced. The cross-sectional area of each mitochondrion, as well as the total cross-sectional area of mitochondria and the total area within each field, were quantified using ImageJ software (version 1.54g, National Institutes of Health, Bethesda, MD, USA). Mitochondrial content was calculated as the ratio of the total cross-sectional area of mitochondria to the total area of the field, and mitochondrial area was calculated as the mean cross-sectional area of the individual mitochondria analyzed within each field. All image analyses were performed by an investigator blinded to genotype. For TEM analysis, the biological “n” refers to the number of independent animals per group. The five fields captured per animal, as well as the individual mitochondria analyzed within each field, were treated as technical replicates; statistical analyses were performed using the values averaged per animal.

### 4.13. Quantification and Statistics

All data were expressed as means ± standard errors of the mean (SEM). Normality of the data was assessed using the Shapiro–Wilk test in combination with visual inspection of data distributions. For datasets that passed the normality test, two-group comparisons were performed using a two-tailed Student’s *t*-test, with paired or unpaired designs as appropriate. For datasets that violated the normality assumption, two-group comparisons were performed using the Mann–Whitney U test. For comparisons among three or more groups, one-way ANOVA followed by Dunnett’s multiple comparison test was applied, as indicated in the figure legends. For the ISO challenge experiment in Tau-Tg mice and the Mdivi-1 intervention study, two-way ANOVA followed by Tukey’s multiple comparison test was used to assess the main effects and interactions. Survival curves were analyzed using the Kaplan–Meier method with the log-rank (Mantel–Cox) test. All statistical analyses were performed using GraphPad Prism software (version 10.1.2, GraphPad Software, San Diego, CA, USA). The value of *p* < 0.05 was considered statistically significant. * *p* < 0.05, ** *p* < 0.01, *** *p* < 0.001, **** *p* < 0.0001.

## Data Availability

Data is contained within the article and Supplementary Materials. The raw RNA-seq data generated in this study have been deposited in the NCBI Gene Expression Omnibus (GEO) under accession number GSE346287.

## Supplementary Materials

The following supporting information can be downloaded at: https://www.mdpi.com/article/10.3390/ijms27188075/s1.
