# Supplementary material for: Tau Overexpression Causes Cardiac Hypertrophy by Disrupting Mitochondrial Homeostasis
Source: Int J Mol Sci. 2026 Sep 11;27(18):8075. doi: 10.3390/ijms27188075 (PMC13607944; doi:10.3390/ijms27188075)
Supplement: Supplementary file 1 [file ijms-27-08075-s001.zip › Supplementary_Data_ijms-4519276.pdf]

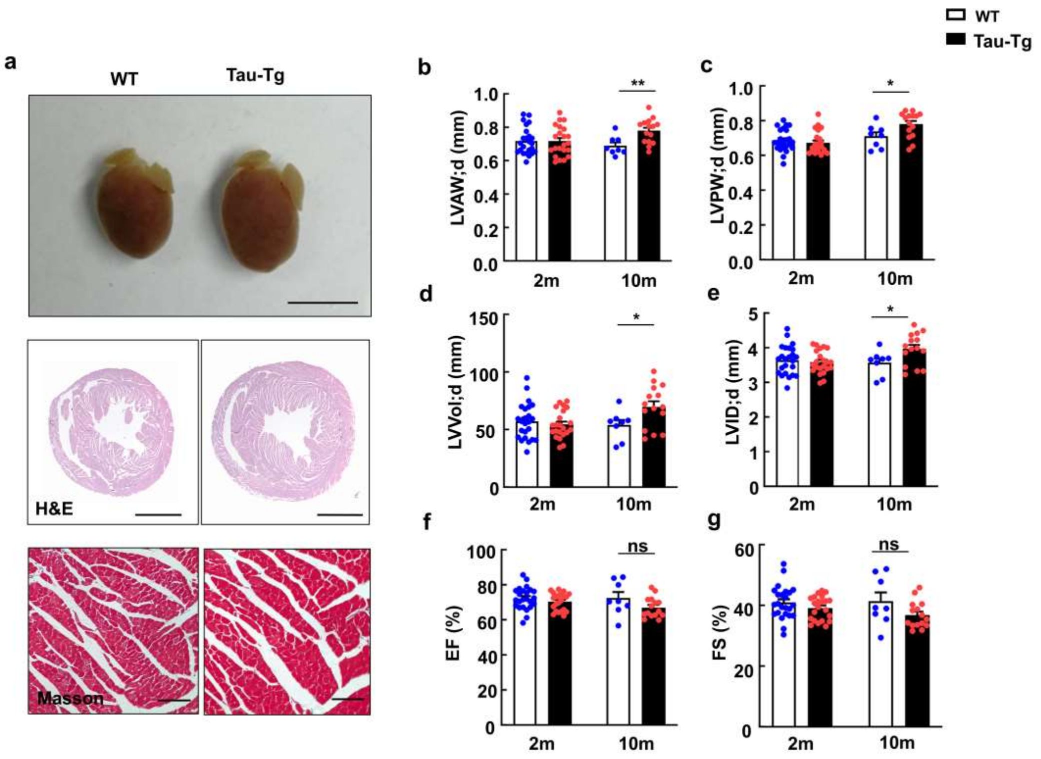

Figure S1 Tau overexpression promotes progressive cardiac remodeling. a Gross and histological morphology of the hearts from different groups of mice. Upper panels: representative gross morphology images of hearts, Scale bar: 5 mm; Middle panels: representative Hematoxylin and Eosin (H&E)-stained cardiac sections from different groups of mice. Scale bar, 1 mm; Lower panels: representative Masson's staining of heart tissue sections, Scale bar: 50 μm. **b-g** Measurement of LVAWd, LVPWd, LVVOLd, LVIDd, EF and FS in different groups of mice. (2-month Control mice: n = 24; 2-month Tau-Tg mice: n = 22, 10-month Control mice: n = 8; 10-month Tau-Tg mice: n = 15). For LVPWd - 10m, statistical significance was determined by Mann - Whitney U test. For LVAWd, Lvvold, Lvidd, EF and FS, statistical significance was determined by Student's t-test. Data are presented as mean ± SEM, **P*<0.05, ***P*<0.01.

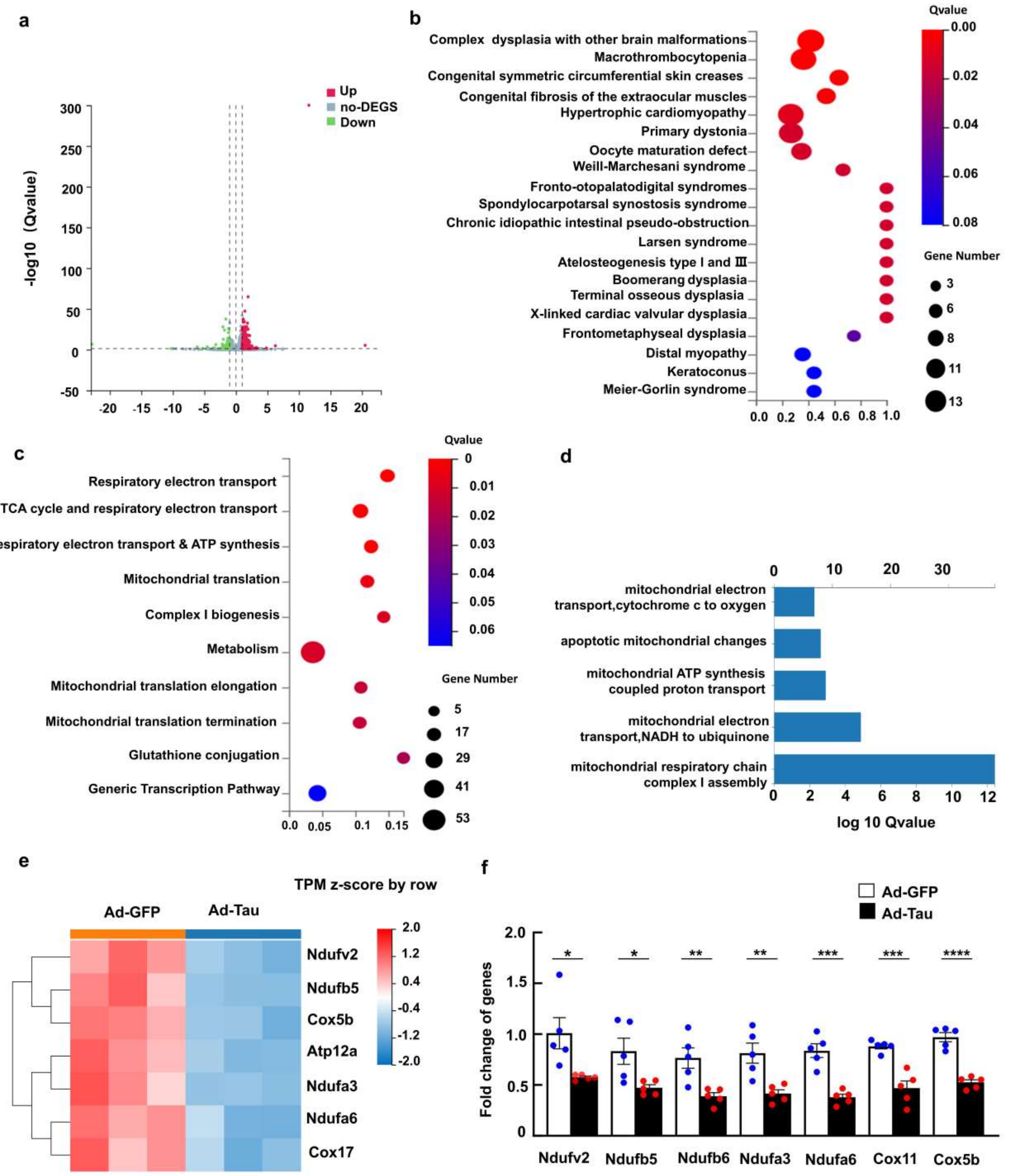

Figure S2 Tau overexpression induces alteration of genes-related with mitochondria homeostasis.

a The volcano plot in RNA-seq analysis. The blue dot indicated the downregulated genes, while the red indicated the upregulated. **b** KEGG analysis of upregulated genes in cardiomyocytes. **c** Enrichment analysis of Reactome pathway of down-regulated genes in cardiomyocytes. **d** GO analysis shows significant downregulation of subunit expression in mitochondrial complexes I and IV assembly and core catalytic reaction pathways. **e** Heatmap of downregulated genes related to mitochondrial complexes I and IV. **f** Real-time PCR verification of changes in mitochondrial complex I and IV core subunit expression (n = 5 independent experiments). Data are presented as mean ± SEM. Statistical significance was calculated by Student's t-test. **P*<0.05, ***P*<0.01, ****P*<0.001, *****P*<0.0001.

Table S1 M-mode echocardiographic characterization of Tau transgenic mice

	2 m		10 m		17 m	
	WT<br>n = 24	Tau-Tg<br>n = 22	WT<br>n = 8	Tau-Tg<br>n = 15	WT<br>n = 8	Tau-Tg<br>n = 15
LV Vol;s (μL)	16.31 ± 1.43	16.42 ± 1.14	15.75 ± 2.81	23.74 ± 2.27	13.95 ± 3.91	58.74 ± 11.87*
LV Vol;d (μL)	57.09 ± 3.13	54.43 ± 2.48	53.84 ± 4.19	69.84 ± 4.60*	50.61 ± 10.01	100.08 ± 11.55*
LVID;s (mm)	2.16 ± 0.08	2.19 ± 0.06	2.11 ± 0.16	2.52 ± 0.11*	1.86 ± 0.27	3.51 ± 0.28**
LVID;d (mm)	3.64 ± 0.08	3.58 ± 0.07	3.56 ± 0.12	3.97 ± 0.11*	3.37 ± 0.29	4.57 ± 0.21**
LVPW;s (mm)	1.18 ± 0.02	1.19 ± 0.03	1.17 ± 0.04	1.24 ± 0.03	1.54 ± 0.16	1.32 ± 0.10
LVPW;d (mm)	0.69 ± 0.01	0.67 ± 0.01	0.71 ± 0.02	0.78 ± 0.02*	1.15 ± 0.16	1.05 ± 0.12
LVAW;s (mm)	1.21 ± 0.02	1.22 ± 0.02	1.14 ± 0.03	1.24 ± 0.03	1.77 ± 0.22	1.40 ± 0.12
LVAW;d (mm)	0.72 ± 0.02	0.72 ± 0.02	0.69 ± 0.02	0.78 ± 0.02**	1.13 ± 0.16	1.02 ± 0.10
EF (%)	72.24 ± 1.28	70.35 ± 1.03	72.45 ± 3.17	67.04 ± 1.44	76.32 ± 5.23	47.70 ± 4.50**
FS (%)	40.92 ± 1.08	39.13 ± 0.81	41.41 ± 2.67	36.82 ± 1.09	46.69 ± 5.01	24.76 ± 2.74**

*P < 0.05, **P < 0.01 and ***P < 0.001 versus respective control mice. Data are presented as mean ± SEM. Statistical significance was determined by Student's t-test.

Table S2 Primer sequences used in this study

Gene	primer sequences
Ndufv2-Rat-s	5’-TCAGTGGGGAAGACATGCAA-3’
Ndufv2-Rat-a	5’-TGGAGTATCTGGGTTATTCTCAGGA-3’
Ndufa6-Rat-s	5’-ACCTCGGTGAAGCCCATTTT-3’
Ndufa6-Rat-a	5’-ATCCCGTCCTTGTTTCACCG’
Ndufb5-Rat-s	5’-TTTCCGAAGACTGTCGCTCC-3’
Ndufb5-Rat-a	5’-TGTTCACCAGTGTTATGCCG’
Ndufa3-Rat-s	5’-ACTGTCTGGGGCCTCGCTAT-3’
Ndufa3-Rat-a	5’-CTCTCACAGGCACTGGGTAGT’
Ndufb6-Rat-s	5’-TACCGCACCAGTCTCTTCAC-3’
Ndufb6-Rat-a	5’-CTGGGCTTCGTGCCAACAAT-3’
GAPDH-Rat-s	5’-TGCCCAGAACATCATCCCT-3’
GAPDH-Rat-a	5’-GGTCCTCAGTGTAGCCCAAG-3’
Cox17-Rat-s	5’-CTCAGGAGAAGAAGCCTCTG-3’
Cox17-Rat-a	5’-CTTTCTCAATGATGCACGCA-3’
Cox5b-Rat-s	5’-GCTTCAAGGTTACTTCGCGG-3’
Cox5b-Rat-a	5’-GTCCCCTCTGTGCTGCTATC-3’
Nppa-Rat-s	5’-ATCTGATGGATTTCAAGAACC-3’
Nppa-Rat-a	5’-CTCTGAGACGGGTTGACTTC-3’
Nppb-Rat-s	5’-TGATTCTGCTCCTGCTTTTC-3’
Nppb-Rat-a	5’-GTGGATTGTTCTGGAGACTG-3’
GAPDH-Mus-s	5’-TGCCCAGAACATCATCCCT-3’
GAPDH-Mus-a	5’-GGTCCTCAGTGTAGCCCAAG-3’
Tau-Mus-s	5’- CTCGCCAGGAGTTTGACACA-3’
Tau-Mus-a	5’- GTCTCCGATGCCTGCTTCTT-3’
Nppa-Mus-s	5’-GCCGGTAGAAGATGAGGTCA-3’
Nppa-Mus-a	5’-GGGCTCCAATCCTGTCAATC-3’
Nppb-Mus-s	5’-GCTCTTGAAGGACCAAGGCCTCAT-3’
Nppb-Mus-a	5’-GATCCGATCCGGTCTATCTTGTGC-3’
Col1a1-Mus-s	5’-AGCGAAGAACTCATACAGCCG-3’
Col1a1-Mus-a	5’-TTGGAGCAGCCATCGACTAG-3’
Col3a1-Mus-s	5'-GCCTCCCAGAACATTACATACC-3'
Col3a1-Mus-a	5'-GGGTAGTCTCATTGCCTTGC-3'

Table S3 Antibodies used for Western blotting in this study

Target antigen	Vendor or Source	Catalog #	Dilution ratio	City	State	Country
GAPDH	ZSGB-BIO	TA-08	1:2000	Beijing	-	China
Tau	Cell Signaling Technology	4019	1:1000	Danvers	MA	USA
Tau-5	Invitrogen	AHB0042	1:1000	Carlsbad	CA	USA
p-Tau	Cell Signaling Technology	9623	1:1000	Danvers	MA	USA
TOMM20	Abcam	ab186734	1:1000	Cambridge	-	UK
p-DRP1	Abcam	ab314755	1:1000	Cambridge	-	UK
DRP1	Abcam	ab184247	1:1000	Cambridge	-	UK
cleaved Caspase-3	ImmunoWay	YM3431	1:1000	Beijing	-	China
Caspase-3	Cell Signaling Technology	9662	1:1000	Danvers	MA	USA
OPA1	Proteintech	27733-1-AP	1:5000	Rosemont	IL	USA
