# Supplementary material for: Tau Overexpression Causes Cardiac Hypertrophy by Disrupting Mitochondrial Homeostasis
Source: Int J Mol Sci. 2026 Sep 11;27(18):8075. doi: 10.3390/ijms27188075 (PMC13607944; doi:10.3390/ijms27188075)

Full unedited images of Western Blot membranes in:

“Tau overexpression causes cardiac hypertrophy by disrupting mitochondrial homeostasis”

Full unedited gel for Fig 1 [a]:

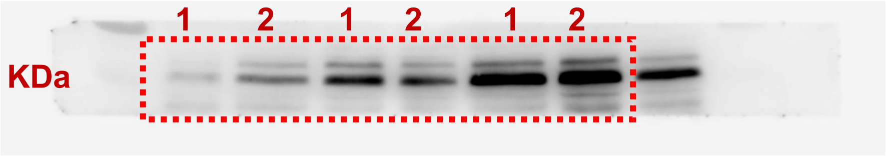

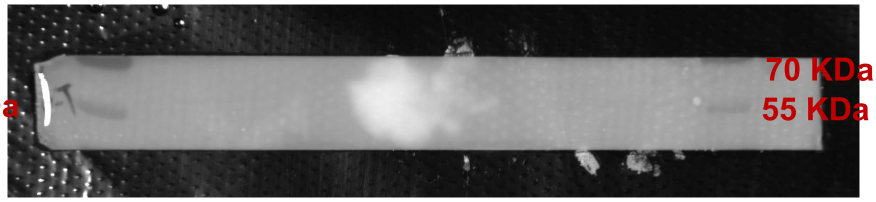

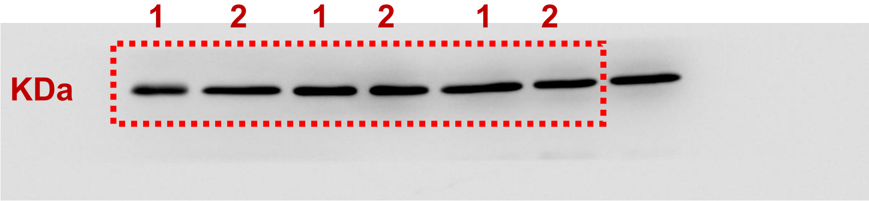

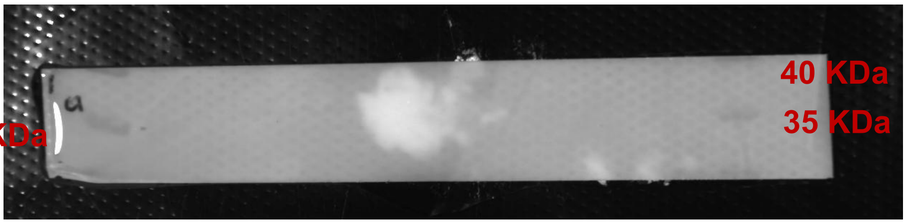

Full unedited gel for Fig 1 [b]:

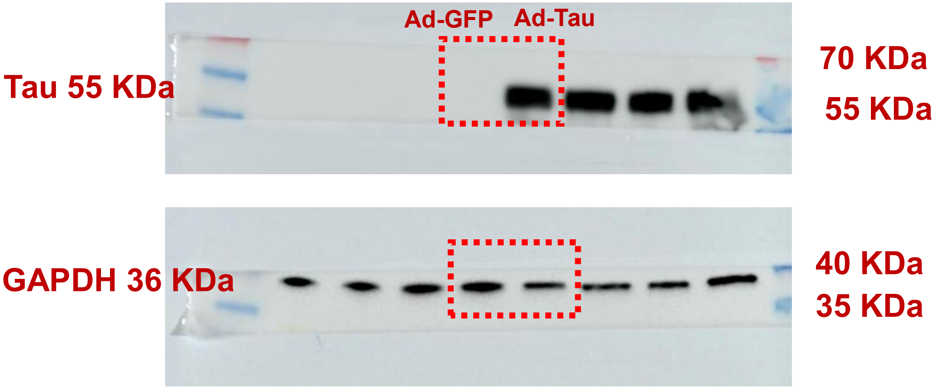

Full unedited gel for Fig 2 [a]:

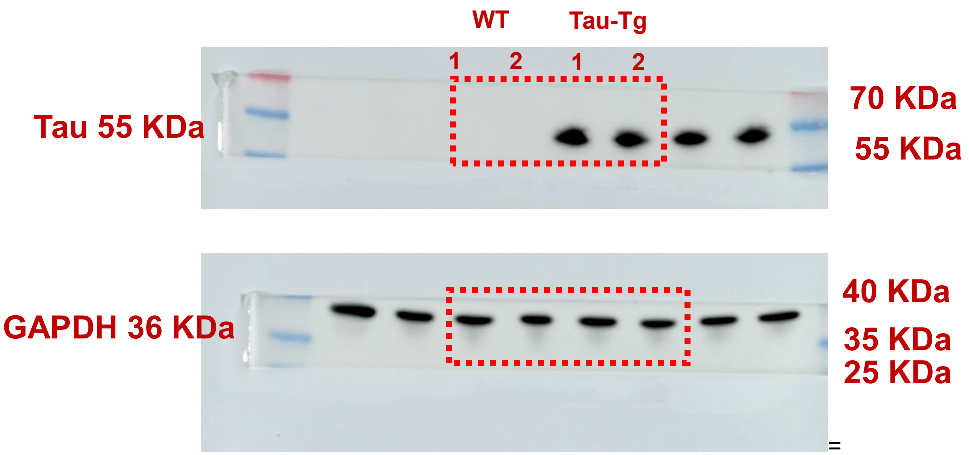

Full unedited gel for Fig 5 [h]:

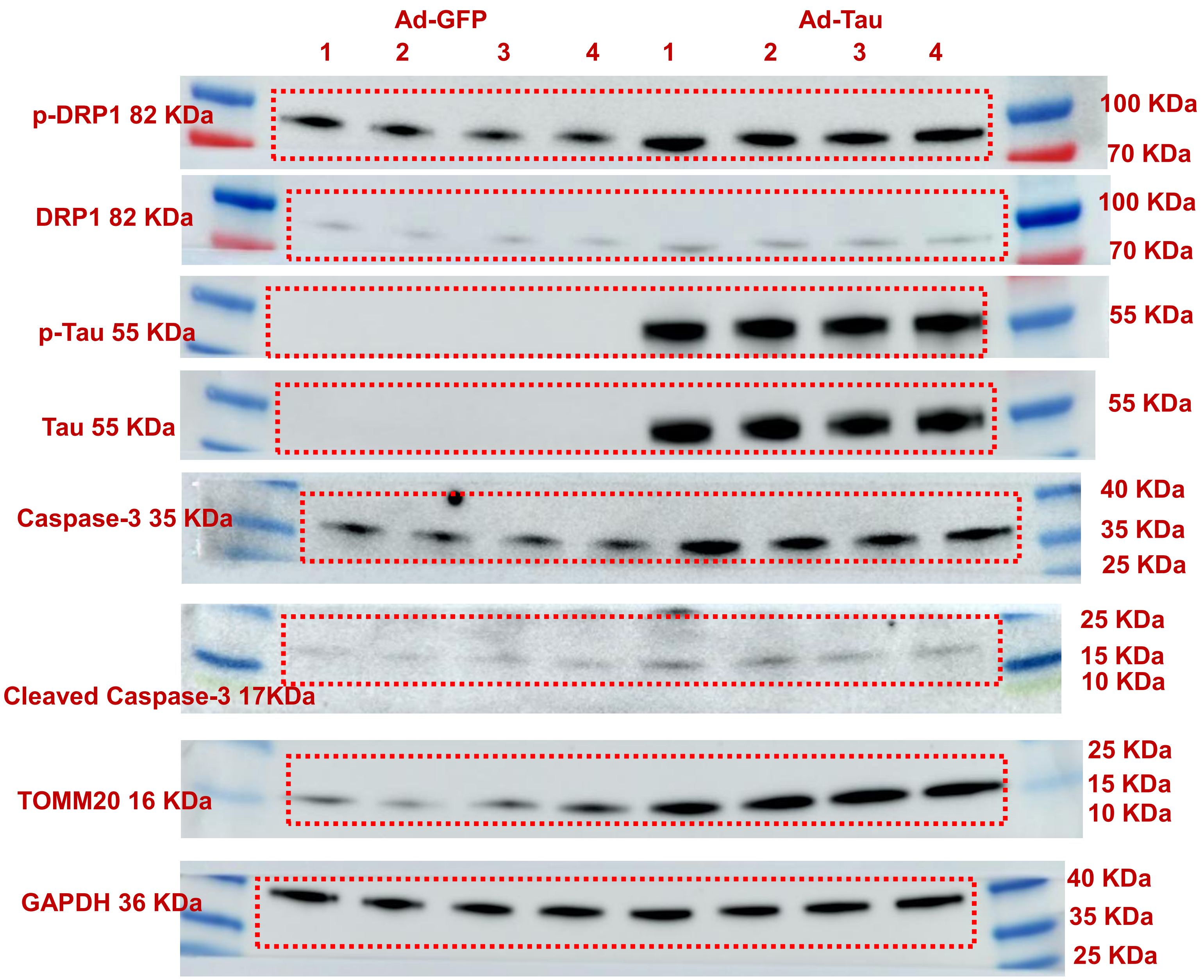

Full unedited gel for Fig 6 [e]:

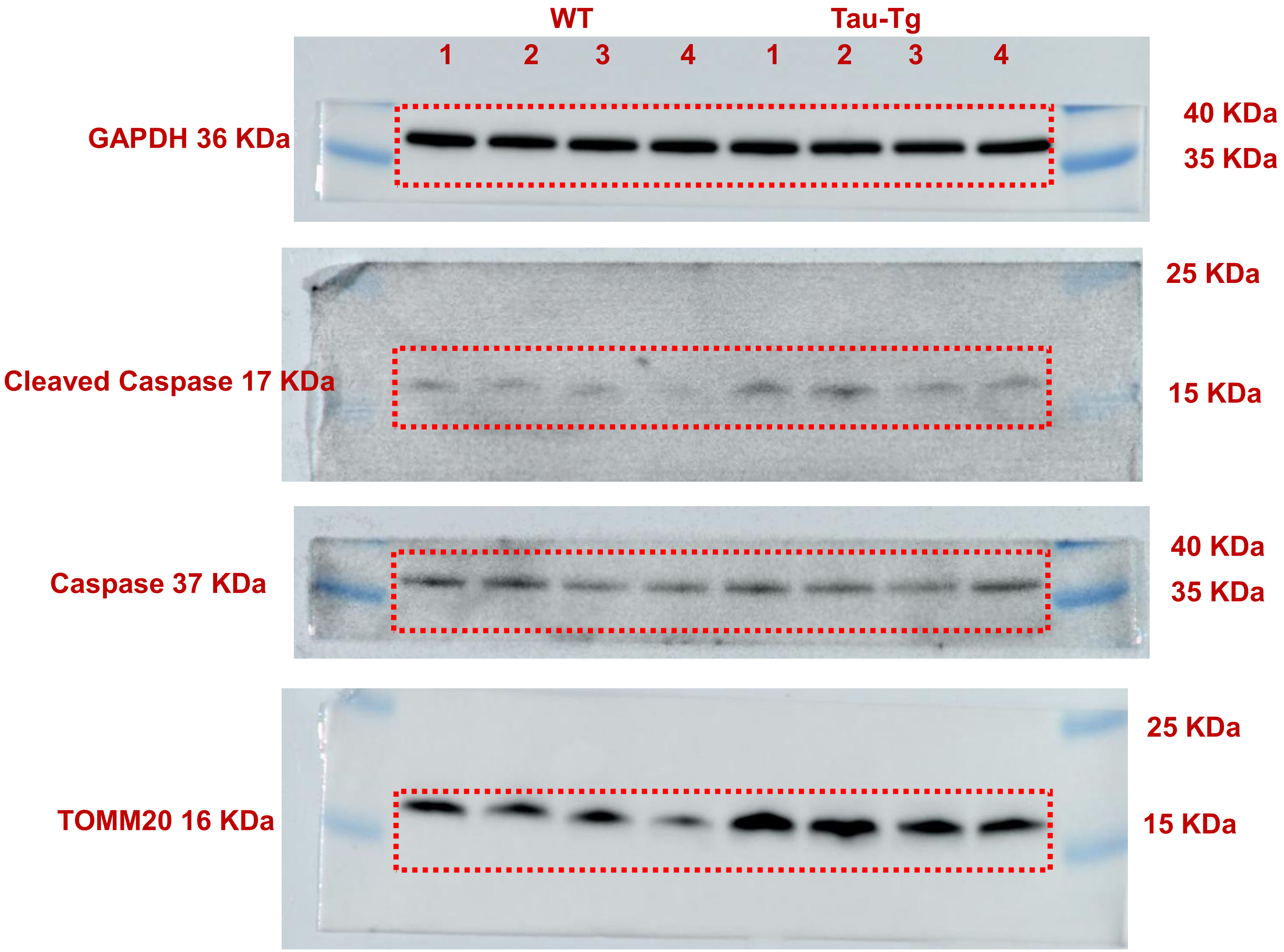

Full unedited gel for Fig 6 [e]:

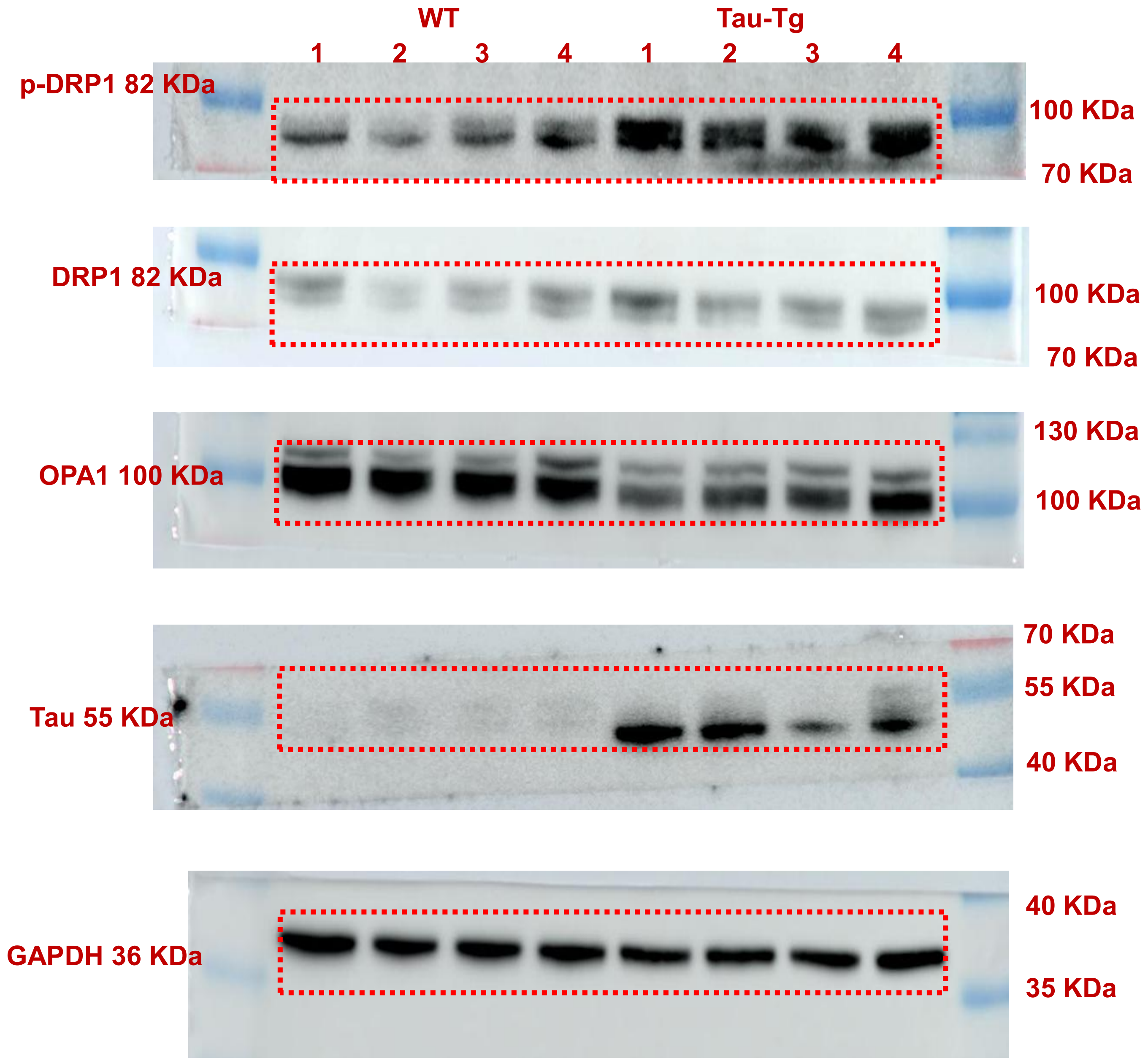

Full unedited gel for Fig 7 [a]:

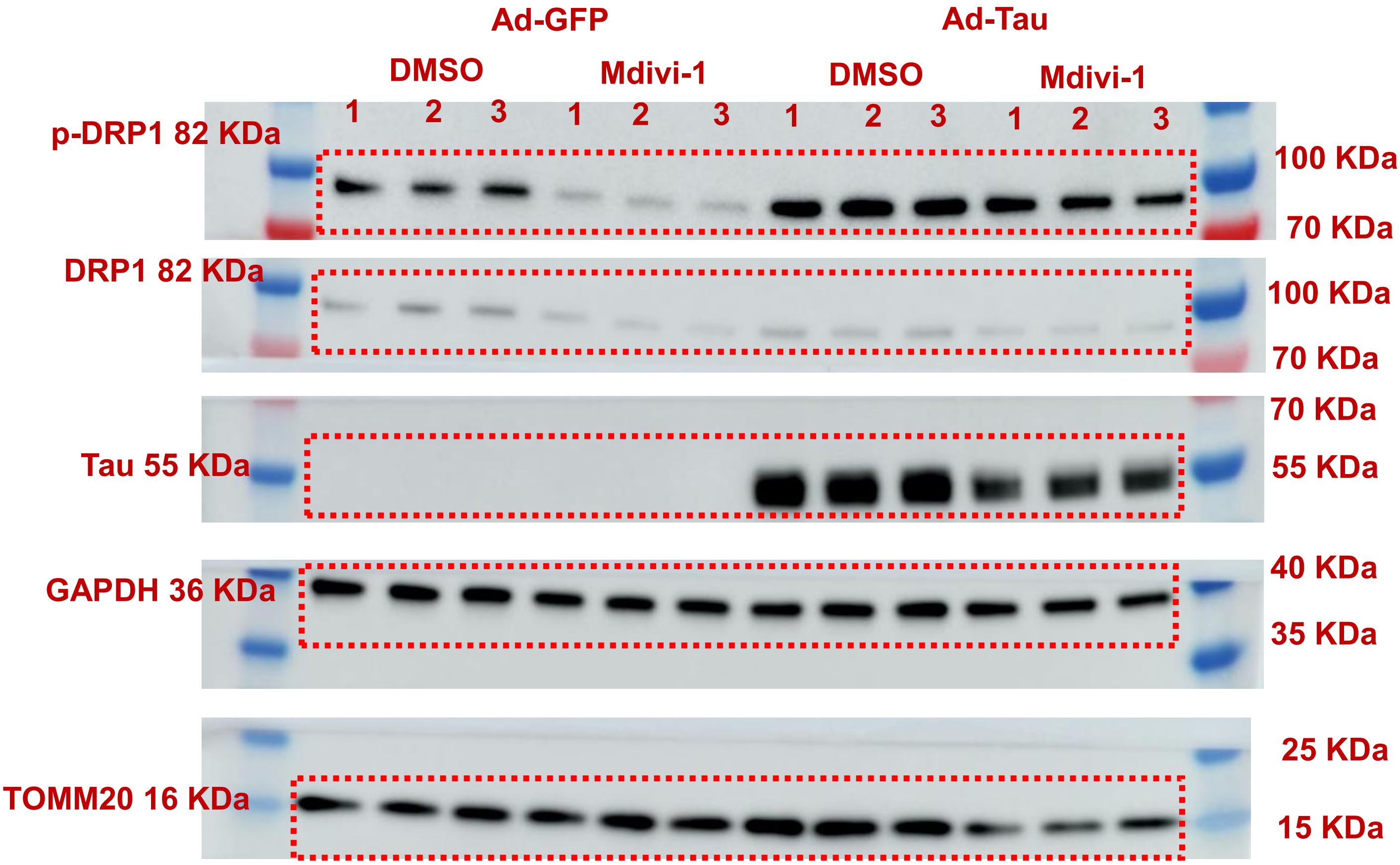

Supplement: Supplementary file 1 [file ijms-27-08075-s001.zip › WB-ijms-4519276.pdf]
